# Efficient clustered, regularly interspaced short palindromic repeats-based gene activation using combinatorial human transcription activation domains

**DOI:** 10.1093/procel/pwaf061

**Published:** 2025-08-25

**Authors:** Yi-Lian Zhou, Yetong Sang, Lingjie Xu, Chuanhong Ren, Weikang Meng, Yu Zhang, Hongqing Liang, Zehua Bao

**Affiliations:** Key Laboratory of Biomass Chemical Engineering of Ministry of Education, College of Chemical and Biological Engineering, Zhejiang University, Hangzhou 310058, China; Zhejiang Key Laboratory of Intelligent Manufacturing for Functional Chemicals, ZJU-Hangzhou Global Scientific and Technological Innovation Center, Zhejiang University, Hangzhou 311215, China; Institute of Bioengineering, College of Chemical and Biological Engineering, Zhejiang University, Hangzhou 310058, China; Key Laboratory of Biomass Chemical Engineering of Ministry of Education, College of Chemical and Biological Engineering, Zhejiang University, Hangzhou 310058, China; Zhejiang Key Laboratory of Intelligent Manufacturing for Functional Chemicals, ZJU-Hangzhou Global Scientific and Technological Innovation Center, Zhejiang University, Hangzhou 311215, China; Institute of Bioengineering, College of Chemical and Biological Engineering, Zhejiang University, Hangzhou 310058, China; Key Laboratory of Biomass Chemical Engineering of Ministry of Education, College of Chemical and Biological Engineering, Zhejiang University, Hangzhou 310058, China; Zhejiang Key Laboratory of Intelligent Manufacturing for Functional Chemicals, ZJU-Hangzhou Global Scientific and Technological Innovation Center, Zhejiang University, Hangzhou 311215, China; Institute of Bioengineering, College of Chemical and Biological Engineering, Zhejiang University, Hangzhou 310058, China; Key Laboratory of Biomass Chemical Engineering of Ministry of Education, College of Chemical and Biological Engineering, Zhejiang University, Hangzhou 310058, China; Zhejiang Key Laboratory of Intelligent Manufacturing for Functional Chemicals, ZJU-Hangzhou Global Scientific and Technological Innovation Center, Zhejiang University, Hangzhou 311215, China; Institute of Bioengineering, College of Chemical and Biological Engineering, Zhejiang University, Hangzhou 310058, China; Institute of Medical Genetics and Development, Key Laboratory of Reproductive Genetics (Ministry of Education) and Department of Reproductive Endocrinology, Women’s Hospital, School of Medicine, Zhejiang University, Hangzhou 310006, China; Key Laboratory of Biomass Chemical Engineering of Ministry of Education, College of Chemical and Biological Engineering, Zhejiang University, Hangzhou 310058, China; Zhejiang Key Laboratory of Intelligent Manufacturing for Functional Chemicals, ZJU-Hangzhou Global Scientific and Technological Innovation Center, Zhejiang University, Hangzhou 311215, China; Institute of Bioengineering, College of Chemical and Biological Engineering, Zhejiang University, Hangzhou 310058, China; Institute of Medical Genetics and Development, Key Laboratory of Reproductive Genetics (Ministry of Education) and Department of Reproductive Endocrinology, Women’s Hospital, School of Medicine, Zhejiang University, Hangzhou 310006, China; Key Laboratory of Biomass Chemical Engineering of Ministry of Education, College of Chemical and Biological Engineering, Zhejiang University, Hangzhou 310058, China; Zhejiang Key Laboratory of Intelligent Manufacturing for Functional Chemicals, ZJU-Hangzhou Global Scientific and Technological Innovation Center, Zhejiang University, Hangzhou 311215, China; Institute of Bioengineering, College of Chemical and Biological Engineering, Zhejiang University, Hangzhou 310058, China; Zhejiang Key Laboratory of Smart Biomaterials, College of Chemical and Biological Engineering, Zhejiang University, Hangzhou 310058, China

## Dear Editor,

Artificial transcription factors (ATFs), which are composed of programmable DNA-binding proteins and transcription activation domains (TADs), have revolutionized the synthetic transcriptional control of genes (Sang et al., 2024). The clustered, regularly interspaced short palindromic repeats (CRISPR) systems have been repurposed as ATFs (Tanenbaum et al., 2014). The resulting CRISPR activators are composed of TADs and a catalytically ‘dead’ CRISPR-associated protein (dCas) complexed with a single guide RNA (sgRNA), which targets a specific gene. Various variants, including dCas-TAD fusions (e.g., dCas9-VP64 and dCas9-VPR), CRISPR-directed synergistic activation mediator, and a signal amplifying dCas9-SunTag system (Chavez et al., 2015; Konermann et al., 2015; Pickar-oliver and Gersbach, 2019; Tanenbaum et al., 2014) have been engineered. While much effort has been devoted to mining and engineering the dCas:sgRNA DNA binding platform, TAD engineering is also crucial for optimizing CRISPR activators. The most potent TADs to date are largely variants of the VP64 domain (a tetramer of the core domain of the simplex herpesvirus virion protein VP16). The current benchmark TAD, VPR, is composed of three TADs, including VP64, p65 (a human NF-κB transcription factor), and Rta (a human herpesvirus replication and transcription activation protein). Previous studies have highlighted that VP16 and Rta inhibit innate immune responses during viral infections (Xing et al., 2013; Zhao et al., 2015; Zheng and Su, 2017). These viral sequences may complicate the advancement of CRISPR activators into clinical translation due to concerns about immunogenicity (Sang et al., 2024). The use of human-derived TADs (hTADs) may minimize such immunogenic potential. Recent progress in the characterization of human transcription factors and the engineering of hTADs provided more options for developing CRISPR activators. Alerasool et al. identified approximately 250 transcription factors by screening the human ORFeome, determined the TADs of 75 transcription factors, and identified several potent TADs, including CITED1-TAD, CITED2-TAD, and CSRNP1-TAD, using a synthetic EGFP reporter assay (Alerasool et al., 2022). DelRosso et al. screened over 2,000 human transcription factors and chromatin regulators using the rTetR DNA binding domain targeted to a synthetic minimal promoter, and annotated 374 activation domains, including the strong MYB-TAD and KLF7-TAD (DelRosso et al., 2023). Using dCas9 targeted to endogenous genes, Tycko et al. identified three potent TADs from human proteins NCOA3, FOXO3, and ZNF473, which were fused to yield a compact TAD named NFZ, with higher transcription activation efficacies than VP64 (Tycko et al., 2020, 2025). The recently reported dCas9-DREAM system fuses TADs derived from human proteins MRTF-A, STAT1, and eNRF2 (MSN) to the bacteriophage coat protein MCP, resulting in the efficient activation of human endogenous genes (Mahata et al., 2023). Although these TADs represent the most potent hTADs to date, there has not been a side-by-side comparison of their transcription activation effects. Whether these human TADs can be further engineered to induce transcription activation levels comparable to those of VPR also remains underexplored.

**Figure 1.**
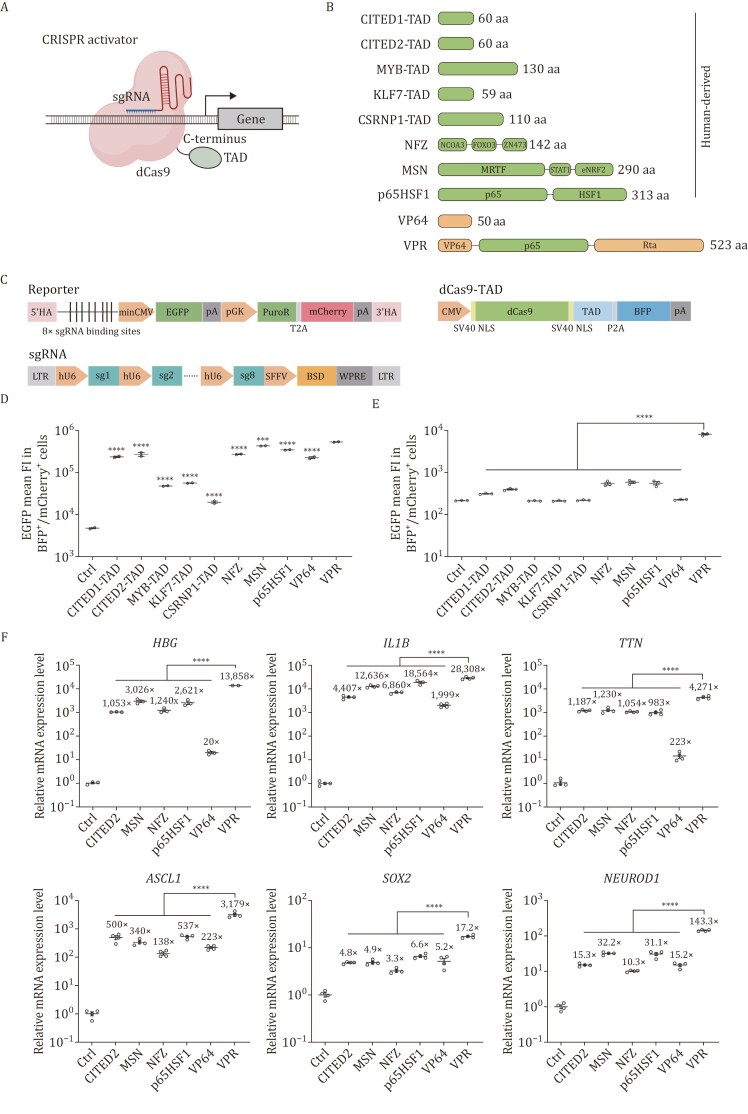
Comparison of gene activation efficiencies of human and viral TADs. (A) Schematic of C-terminal fused dCas9-TAD ATF used in this study. (B) Architectures and sizes of human and viral TADs. VP64 and Rta are viral protein sequences. (C) Expression constructs of the EGFP reporter, sgRNAs, and dCas9-TAD. 5′HA/3′HA: 5′ homology arm/3′ homology arm; sgRNA: single guide RNA; minCMV: minimal cytomegalovirus promoter; EGFP: enhanced green fluorescent protein; pA: polyadenylation signal; pGK: phosphoglycerate kinase promoter; PuroR: puromycin resistance; T2A: thosea asigna virus 2A peptide; mCherry: monomeric Cherry fluorescent protein; LTR: long terminal repeat; hU6: human U6 promoter; SFFV: spleen focus-forming virus promoter; BSD: blasticidin resistance; WPRE: woodchuck hepatitis virus posttranscriptional regulatory element; CMV: cytomegalovirus promoter; SV40 NLS: simian virus 40 nuclear localization signal; dCas9: dead Cas9; TAD: transcriptional activation domain; P2A: porcine teschovirus-1 2A peptide; BFP: blue fluorescent protein. (D) Plasmid EGFP mean fluorescence intensity in BFP and mCherry dual-positive populations at 48 h post-transfection with different dCas9 fusion protein constructs. Ctrl, dCas9 without TADs. The data were graphed as mean ± SEM and represent two biological replicates. Significance levels were calculated by one-way ANOVA followed by Dunnett’s test against VPR. ***P < 0.001; ****P < 0.0001. (E) Chromosomal EGFP mean fluorescence intensity in BFP and mCherry dual-positive populations at 48 h post-transfection with different dCas9 fusion protein constructs. The reporter cell line contains an *AAVS1* integrated EGFP and lentivirally integrated sgRNAs. Ctrl, dCas9 without TADs. The data were graphed as mean ± SEM and represent three biological replicates. Significance levels were calculated by one-way ANOVA followed by Dunnett’s test against VPR. ****P < 0.0001. (F) Relative expression levels of endogenous human genes in HEK293T after dCas9-CITED2, dCas9-MSN, dCas9-NFZ, dCas9-p65HSF1, dCas9-VP64, and dCas9-VPR were targeted to their respective promoters using pools of four sgRNAs as measured by qRT-PCR. All qRT-PCR samples were collected at 72 h post-transfection. The housekeeping gene *glyceraldehyde phosphate dehydrogenase* (*GAPDH*) was used as an internal control for the normalization of qRT-PCR data. Ctrl, dCas9 without TADs. The data were graphed as mean ± S.E.M and represent at least three biological replicates. Significance levels were calculated by one-way ANOVA followed by Dunnett’s test against VPR. ****P < 0.0001.

To perform side-by-side comparisons of the recently reported hTADs in the same experimental settings, we constructed carboxyl-terminal fusion proteins of dCas9 with eight hTADs (Fig. 1A), including CITED1-TAD, CITED2-TAD, MYB-TAD, KLF7-TAD, CSRNP1-TAD, NFZ, MSN, and p65HSF1 (Konermann et al., 2015; Fig. 1B). We first tested gene activation efficiencies of these hTADs against VP64 and VPR on an EGFP reporter driven by a minimal promoter downstream of eight sgRNA binding sites in HEK293T cells (Fig. 1C). The results showed that the plasmid EGFP activation efficiencies of human-derived p65HSF1, MSN, NFZ, and CITED2-TAD were higher than VP64 (Fig. 1D). We then further tested the chromosomally integrated EGFP activation efficiencies of these TADs. The results confirmed that the activation efficiencies of p65HSF1, MSN, NFZ, CITED2-TAD, and CITED1-TAD were higher than VP64. However, their activation efficiencies were substantially lower than VPR (Fig. 1E). We examined their expression levels, and their lower activation efficiencies were not due to poor expression (Fig. S1). To validate this observation at endogenous loci, we examined the activation efficiencies of p65HSF1, MSN, NFZ, and CITED2-TAD on endogenous gene targets in HEK293T cells. We chose genes that are either therapeutic targets (*HBG*, *IL1B*, and *TTN*) or encode transcription factors for cellular differentiation (*ASCL1*, *SOX2*, and *NEUROD1*). *HBG* (Hemoglobin Gamma) belongs to the β-globin gene cluster and contains two highly homologous genes: *HBG1* and *HBG2*. Interleukin-1 Beta (IL1B), as a pro-inflammatory cytokine, is involved in innate immunity and inflammatory responses. *TTN* encodes titin, which is an important functional component of striated muscle tissues. *ASCL1* and *NEUROD1* encode transcription factors that dictate the fate of neurons, and *SOX2* encodes a transcription factor involved in stem cell self-renewal. These genes are expressed at low basal or undetectable levels in HEK293T cells with closed chromatin states at their promoters, except for *SOX2*, which expresses moderately and has a partially accessible promoter (Fig. S2). Relative mRNA expression levels were examined at three days after transfection of the targeting sgRNAs and dCas9-TAD constructs. Results showed that all four hTADs exerted activation effects on all tested endogenous genes (Fig. 1F). Consistent with the reporter assay, activation effects of hTADs were weaker than VPR on all tested genes, but comparable to VP64 at some of the gene loci. Their relative expression levels were also consistent with their expression levels in the stable EGFP reporter cell line, all higher than VPR (Fig. S3). These results highlight room for further improvement of the hTADs’ efficacies.

**Figure 2.**
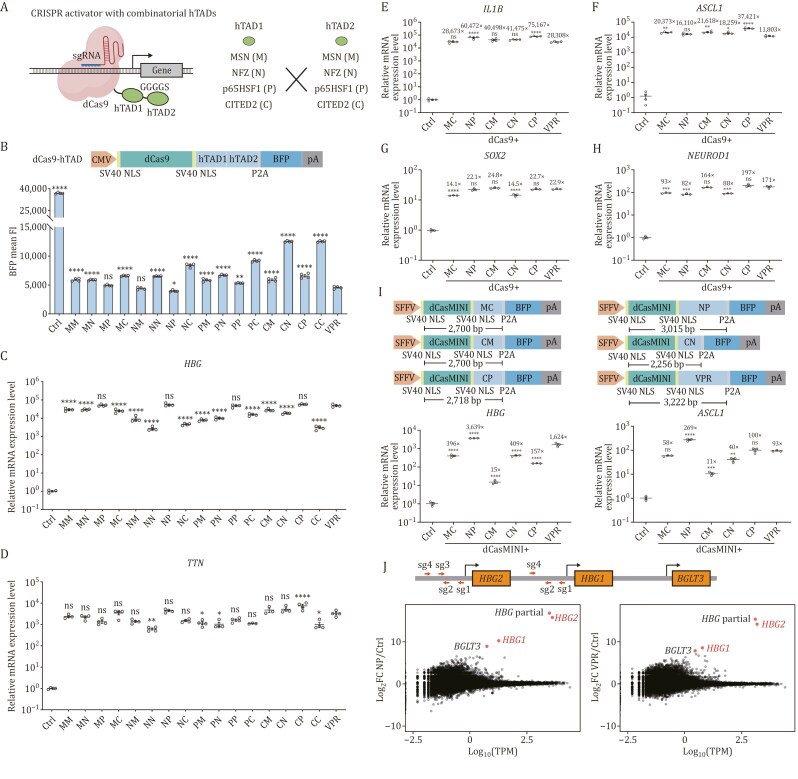
Engineering and evaluation of combinatorial human TADs. (A) Schematic of CRISPR activator with combinatorial hTADs. (B) Expression levels of dCas9-hTAD as measured by BFP mean fluorescence intensity. Ctrl, dCas9 without TADs. The data were graphed as mean ± S.E.M and represent four biological repeats. Significance levels were calculated by one-way ANOVA followed by Dunnett’s test against VPR. *P < 0.05; **P < 0.01; ****P < 0.0001; ns, not significant. (C–H) Relative expression levels of endogenous human genes after dCas9-hTADs were targeted to their respective promoters using pools of four sgRNAs as measured by qRT-PCR. Ctrl, dCas9 without TADs. All qRT-PCR samples were collected at 72 h post-transfection. The housekeeping gene *glyceraldehyde phosphate dehydrogenase* (*GAPDH*) was used as an internal control for the normalization of qRT-PCR data. The data were graphed as mean ± SEM and represent at least three biological repeats. Significance levels were calculated by one-way ANOVA followed by Dunnett’s test against VPR. *P < 0.05; **P < 0.01; ***P < 0.001; ****P < 0.0001; ns, not significant. (I) dCasMINI-based CRISPR activation. Relative expression levels of endogenous human genes after TADs fused with dCasMINI were targeted to their respective promoters as measured by qRT-PCR. Ctrl, dCasMINI without TADs. All qRT-PCR samples were collected at 72 h post-transfection. The housekeeping gene *glyceraldehyde phosphate dehydrogenase* (*GAPDH*) was used as an internal control for the normalization of qRT-PCR data. The data were graphed as mean ± S.E.M and represent three biological repeats. Significance levels were calculated by one-way ANOVA followed by Dunnett’s test against VPR. **P < 0.01; ***P < 0.001; ****P < 0.0001; ns, not significant. (J) Transcriptome profiling of dCas9-NP and dCas9-VPR targeted to the *HBG* locus. HEK293T cells transiently expressing dCas9-NP or dCas9-VPR together with *HBG*-targeting sgRNAs were harvested 72 h post-transfection for RNAseq analysis. *HBG* partial, a truncated *HBG* transcript mapped to both *HBG1* and *HBG2*. Ctrl, dCas9 without TADs. The data represent the mean of two biological replicates. TPM, transcripts per kilobase million. FC, fold change.

The combination of multiple TADs can improve the activation efficacy of ATFs, as in the examples of engineering VPR, NFZ, and MSN (Chavez et al., 2015; Mahata et al., 2023; Tycko et al., 2025). To further improve the efficacy of human TADs, we investigated the possibility of enhanced activation by combining the most potent hTADs identified. We constructed all 16 pairwise fusions of MSN (M), NFZ (N), p65HSF1 (P), and CITED2-TAD (C) (Fig. 2A) and compared their activation effects on two endogenous genes, *HBG* and *TTN*. We first made sure that their transfection efficiencies were similar by spiking in a GFP-expressing plasmid and measuring the percentage of GFP-positive populations (Fig. S4). Without GFP spike-in, we estimated the expression levels of these fusion proteins by measuring the fluorescence intensity of a P2A-fused blue fluorescent protein. We found that most fusions were expressed at a higher level than VPR in HEK293T cells, except for MP, NM, and NP (Fig. 2B). We further selected successfully transfected populations through puromycin selection. The relative expression levels of different hTADs were consistent with or without selection (Fig. S5), indicating that transfection efficiencies were similar between different hTADs. After puromycin selection, the expression levels of MP, NM, and NP improved slightly. We also observed that NC, CN, and CC were expressed substantially higher among the fusions, potentially due to their smaller sizes. We then observed that transcription activation efficiencies of multiple fusions were improved at the *HBG* site (Figs. 2C, S6, and S7), as compared to the individual hTADs (Fig. 1F). Their relative activation efficiencies were consistent with results obtained from GFP spike-in experiments (Fig. S6) or after puromycin selection (Fig. S7). Among these fusions, MSN-CITED2 (MC, 355 aa), NFZ-p65HSF1 (NP, 460 aa), CITED2-MSN (CM, 355 aa), CITED2-NFZ (CN, 207 aa), and CITED2-p65HSF1 (CP, 378 aa) showed consistently high efficacies at both genes (Fig. 2C and 2D; Table S1). The activation effects of these combinatorial hTADs were comparable with VPR, and their sizes are smaller than VPR (523 aa), which may have advantages in gene delivery.

To further evaluate if the improved activation efficacy of the combinatorial hTADs can be generalized to other target genes, we tested the activation effects of dCas9 fused NP, MC, CM, CN, and CP at six additional endogenous genes (*IL1B*, *ASCL1*, *SOX2*, *NEUROD1*, *RHOXF2*, and *NEUROG2*, Fig. S2). For *IL1B*, CP and NP showed the highest activation efficiencies (Fig. 2E). For *ASCL1* and *RHOXF2*, CP showed the highest activation efficiencies (Figs. 2F and S8). For these three genes, all combinatorial hTADs showed comparable or higher activation efficiencies than VPR. For *SOX2*, *NEUROD1*, and *NEUROG2*, CM and CP showed comparable activation efficiencies with VPR (Figs. 2G, 2H, and S8). Overall, despite the target-specific variability, multiple combinatorial hTADs, including NP (higher than or comparable to VPR at four out of six genes), CM (higher than or comparable to VPR at all six genes), and CP (higher than or comparable to VPR at all six genes), seem to be the most potent combinatorial hTADs that are generalizable to different gene targets. In addition, relative potencies were reproducible at longer time points post-transfection (Fig. S9). We then evaluated these hTADs in HeLa cells. Although transfection efficiencies were lower in HeLa cells than in HEK293T cells, transfection efficiencies and expression levels of different combinatorial hTADs were similar (Fig. S10). Targeting *IL1B* and *ASCL1*, MC, CM, and CP had similar activation levels, while NP and particularly CN showed lower activation levels at both genes (Figs. S11 and S12). In human embryonic stem cells (hESCs), we tested their activation levels at *ASCL1* and *NEUROD1*, two genes involved in neuronal lineage differentiation (Fig. S13). At *ASCL1*, MC, CM, and CP were more potent, consistent with data in HEK293T cells (Fig. 2F) and in HeLa cells (Figs. S11 and S12). At *NEUROD1*, MC and CN were more potent, comparable to VPR. In addition, the overall activation fold changes were higher than those in HEK293T cells (Fig. 2H), potentially due to the positive feedback of NeuroD1 in hESCs. To further evaluate the potential utility of these hTADs in different cellular contexts, we compared them in activating an mCherry reporter gene in yeast. The results showed that NP, CM, and CP showed comparable activation efficiencies as VPR (Fig. S14), consistent with the results obtained in HEK293T cells. The above results collectively showed that combinatorial hTADs have improved performance across different cell lines and species. However, it is noted that MC is more efficient than NP in HeLa cells, suggesting potential impacts of cellular environments.

Small Cas systems, such as Cas12f, have smaller sizes than Cas9, making them more suitable for gene delivery. Through engineering the sgRNA structure and directed evolution of the Cas12f protein, a CasMINI system was recently obtained with greatly improved gene editing capability. It was also mutated into a nuclease-inactive dCasMINI when fused with VPR, efficiently activating endogenous gene expression (Xu et al., 2021). To investigate whether our combinatorial hTADs are compatible with a small Cas platform, thus further reducing the ATF size, we fused dCasMINI with the combinatorial hTADs (Fig. 2I) and compared their activation efficiencies against VPR on *HBG* and *ASCL1* in HEK293T cells. We made sure that their transfection efficiencies were similar (Fig. S15). When fused with dCasMINI, we noticed that the expression levels of CM, CN, and MC were lower (Fig. S16). NP retained high activation efficiencies higher than that of VPR on both gene targets (*P* < 0.0001), while CP is comparable to VPR at *ASCL1* (Fig. 2I). With dCasMINI, NP seems to be the best-performing combinatorial hTAD, potentially due to its better expression.

Finally, we benchmarked the gene activation specificity of dCas9-NP against dCas9-VPR. We performed transcriptome sequencing of the *HBG* samples targeted by either dCas9-NP or dCas9-VPR. Three of the four sgRNAs target both *HBG1* and *HBG2* (Figs. 2J and S17). The number of differentially expressed genes as compared to a dCas9-only control was 120 for dCas9-NP and 76 for dCas9-VPR, out of 62,704 examined transcript isoforms (Tables S2 and S3). Among them, the fold changes of *HBG1* and *HBG2* genes targeted by dCas9-NP were 1,206-fold and 55,443-fold (Table S2), respectively, while the fold changes of *HBG1* and *HBG2* genes targeted by dCas9-VPR were 375-fold and 18,345-fold (Table S3), respectively. Overall, only the *HBG* locus-associated transcripts were substantially upregulated by both dCas9-NP and dCas9-VPR (Fig. 2J). We also observed high correlations between NP and VPR transcriptomes, which are similar to the correlations between biological replicates (Table S5). These results indicated that dCas9-NP exhibited high activation efficiencies while not substantially sacrificing targeting specificity.

Recent advancements in the high-throughput discovery of hTADs (Alerasool et al., 2022; DelRosso et al., 2023) and engineering of hTAD fusions (Mahata et al., 2023; Tycko et al., 2025) are exciting. However, our side-by-side comparisons revealed that currently reported hTADs exhibited weaker activation effects when directly fused with dCas9, as compared to VPR (Fig. 1). The combinatorial hTADs developed in this study showed comparable efficiencies as VPR in certain contexts, making them ideal synthetic biology parts for regulating cellular functions while potentially minimizing the risk of immunogenicity. Analysis of these combinatorial hTADs using an immunogenicity prediction tool revealed that they contain less likely immunogenic 9mer peptides (Supplementary Text and Table S4). Nevertheless, validating their true immunogenicity ultimately requires empirical measurements in a clinical setup.

The CRISPR activators developed in this study have simple compositions, consisting of only guide RNAs and the dCas-TAD protein. NP, CM, and CP are all smaller than VPR, while still retaining comparable activating efficiencies. Although CN is weaker at some of the gene loci, it is substantially smaller with only 207 amino acids, which can be used in scenarios where compactness is prioritized, such as when multiple orthogonal dCas proteins are used to regulate complex gene regulatory networks or additional control switches need to be incorporated (Sang et al., 2024). We additionally demonstrated that NP is compatible with a small dCasMINI platform, further improving compactness. We envision that these combinatorial hTADs can be extended to other dCas proteins to further expand the targetable sequence space (Cai et al., 2024).

In summary, we benchmarked hTADs and developed combinatorial hTAD fusions based on the dCas-TAD carboxyl-terminal fusion architecture. We observed improved performance for several combinations, particularly the NFZ-p65HSF1 (NP) chimeric module, which displayed comparable gene activation efficiencies as VPR in certain contexts. In addition, the NP hTAD exhibited high transcriptional activation specificity and is compatible with a small dCas system, making it a promising CRISPRa effector.

## Supplementary data

Supplementary data is available at *Protein & Cell* online https://doi.org/10.1093/procel/pwaf061.

pwaf061_Supplementary_Figures_S1-S17_Tables_S1-S5

## Footnotes

We thank Dr. Xia Liu from Zhejiang University for providing the pLenti-U6-Cas9 plasmid and Dr. Jiazhang Lian from Zhejiang University for providing the yeast strain. We thank Dr. Baohui Chen from Zhejiang University for helpful discussions. We thank iBioFoundry and the Core Facility at the Institute for Intelligent Bio/Chem Manufacturing, ZJU-Hangzhou Global Scientific and Technological Innovation Center for analytical support.

This work was supported by the National Key R&D Program of China (Grant No. 2023YFF1204500 to ZB), the National Natural Science Foundation of China (grant No. 22308316 to ZB), and Fundamental Research Funds for the Central Universities (Grant Nos. 226-2025-00043 and 226-2022-00214 to ZB).

All data are available in the main text or the supplementary materials. All materials are available upon request. The raw reads of the RNA sequencing data were deposited into the Sequence Read Archive database (accession number: PRJNA1245128) at the National Center for Biotechnology Information (NCBI).

Z.B. and Y.L.Z. designed the research. Y.L.Z. and Y.Z. performed the EGFP activation experiments. Y.L.Z., Y.S., C.R., and L.X. benchmarked the individual hTADs and top-performing combinatorial hTADs. Y.L.Z., L.X., and Y.S. benchmarked all the pairwise combinatorial hTADs. Y.S. and L.X. performed the long-term activation experiments. Y.L.Z. and C.R. performed the dCasMINI experiments. Y.L.Z. performed the RNA sequencing experiments. Y.S. performed the HeLa and yeast experiments. W.M., L.X., and H.L. performed experiments in hESCs. Y.L.Z., Y.S., L.X., and C.R. analyzed the data and made the figures. Y.L.Z. and Z.B. wrote the manuscript. Z.B. supervised the research. No AI-assisted technologies were used in any part of this work.
