# Supplementary material for: Efficient clustered, regularly interspaced short palindromic repeats-based gene activation using combinatorial human transcription activation domains
Source: Protein Cell. 2025 Aug 25;17(2):163–9. doi: 10.1093/procel/pwaf061 (PMC12959769; doi:10.1093/procel/pwaf061)
Supplement: pwaf061_Supplementary_Figures_S1-S17_Tables_S1-S5 [file pwaf061_supplementary_figures_s1-s17_tables_s1-s5.pdf]

SUPPLEMENTARY MATERIAL

Efficient clustered, regularly interspaced short palindromic repeats-based gene activation using combinatorial human transcription activation domains

Yi-Lian Zhou,[1,2,3,4,#] Yetong Sang,[1,2,3,#] Lingjie Xu,[1,2,3,#] Chuanhong Ren,[1,2,3,#] Weikang Meng,[5] Yu Zhang,[1,2,3] Hongqing Liang,[5] and Zehua Bao[1,2,3,6,★]

[1]Key Laboratory of Biomass Chemical Engineering of Ministry of Education, College of Chemical and Biological Engineering, Zhejiang University, Hangzhou 310058, China

[2]Zhejiang Key Laboratory of Intelligent Manufacturing for Functional Chemicals, ZJU-Hangzhou Global Scientific and Technological Innovation Center, Zhejiang University, Hangzhou 311215, China

[3]Institute of Bioengineering, College of Chemical and Biological Engineering, Zhejiang University, Hangzhou 310058, China

[4]Current address: Institute of Hydrobiology, Zhejiang Academy of Agricultural Sciences, Hangzhou 310021, China

[5]Institute of Medical Genetics and Development, Key Laboratory of Reproductive Genetics (Ministry of Education) and Department of Reproductive Endocrinology, Women's Hospital, School of Medicine, Zhejiang University, Hangzhou 310006, China

[6]Zhejiang Key Laboratory of Smart Biomaterials, College of Chemical and Biological Engineering, Zhejiang University, Hangzhou 310058, China

[#]These authors contributed equally.

[★]Correspondence should be addressed to Z.B. (zbao@zju.edu.cn).

MATERIALS AND METHODS

Cell culture

HEK293T (Cat. # GNHu44) cells and HeLa cells (Cat. # TCHu187) were purchased from the National Collection of Authenticated Cell Cultures (Shanghai, China) and cultured in DMEM with high glucose, sodium pyruvate, and GlutaMAX (Gibco, USA), additionally supplemented with 10% FBS (ExCell Bio, China). Cells were grown at 37 °C under 5% CO_2 in a humidified incubator and maintained at confluency below 90%. The hESC line H1 was cultured in mTeSR1 medium (StemCell Technologies, Cat. # 85851) on matrigel-coated plates (Corning, Cat. # 354277) at 37 °C under 5% CO_2 in a humidified incubator. H1 cells were passaged at 1:5 ratio every 4-5 days upon 5 minutes treatment using Gentle Cell Dissociation Reagent (StemCell Technologies, Cat. # 100-0485).

Generation of the reporter cell line

The EGFP reporter construct with flanking homology arms targeting the *AAVS1* site was commercially synthesized (Jiutian Gene Technology, Tianjin, China) according to the sequences of pMP472 (Addgene #134997) and cloned as a plasmid. The *AAVS1* targeting CRISPR/Cas9 plasmid (pLenti-U6-AAVS1 sgRNA-Cas9) was constructed by inserting an AAVS1 sgRNA into the plasmid backbone (pLenti-U6-Cas9, a gift from Dr. Xia Liu, Zhejiang University). The EGFP reporter plasmid was co-transfected with pLenti-U6-AAVS1 sgRNA-Cas9 into HEK293T cells to perform chromosomal integration. Three days after transfection, cells were transduced with lentiviruses expressing eight pre-screened sgRNAs targeting the binding sites upstream of the reporter. Transduced cells were selected with 2 μg/mL puromycin

(Gibco, USA) and 10 μg/mL blasticidin (Gibco, USA) for ten days with passaging every 3-4 days. Survived cells were sorted into mCherry-positive single clones using a flow cytometer (BD FACSAria™ III, USA). One clone displaying a single mCherry peak was chosen. The correct insertion of the reporter sequence into the *AAVS1* site was validated by genomic PCR. This clone was further cultured to establish a cell line for subsequent experiments.

Plasmid construction

Plasmids were cloned by standard molecular cloning techniques. pAC1410 (Addgene #71907) was used as the backbone plasmid for dCas-TAD constructs. VP64 and VPR gene fragments were amplified from pRS415-Cas9-VPR (Addgene #163971). P65-HSF1 gene fragment was amplified from pAC1410. Gene fragments encoding KLF7-TAD, MYB-TAD, CSRNP1-TAD, CITED1-TAD, CITED2-TAD, MSN, and NFZ were human codon-optimized and commercially synthesized (Jiutian Gene Technology, Tianjin, China). sgRNA expression plasmids were constructed by ligating the corresponding annealed oligos to the backbone plasmid pGL3-U6-gRNA-BSD (derived by replacing the EGFP gene of Addgene #107721 with a blasticidin S deaminase gene marker) downstream of the human U6 promoter. For puromycin selection of successfully transfected cells, the sgRNA expression backbone was derived by deleting the Cas9 expression cassette of Addgene #98292 and further replacing the neomycin resistance marker with a puromycin resistance marker. dCasMINI fragment was commercially synthesized (Jiutian Gene Technology, Tianjin, China) according to pSLQ9926 (Addgene # 176269). The plasmids used for yeast mCherry activation were constructed using pCRCT (Addgene #60621) as the backbone. The Cas9 fragment was replaced by dCas9. The *URA3*

selection marker was replaced by *LEU2*, which was amplified from p415-GalL-Cas9-CYC1t (Addgene #43804). The different TAD fragments (VPR, MC, NP, CM, CN, and CP) were amplified from corresponding pAC1410-dCas9-TAD plasmids and inserted between the NLS at the C-terminus of dCas9 and the ADH2 terminator. The sgRNA sequences were synthesized as oligos, annealed, and cloned into the backbone of each pCRCT-dCas9-TAD plasmid. The sequences of all sgRNAs are listed in Supplementary **Table S6**.

Transfection

All HEK293T and HeLa cells were transfected with polyethyleneimine (PEI, YEASEN, China) unless otherwise noted with Lipofectamine 5000 (Cat. # L3200, Solarbio, China). The total amount of plasmids was 500 ng per well for a 24-well plate. Approximately 2×10^5 cells were plated per well one day before transfection. For plasmid EGFP activation experiments, plasmids encoding the EGFP reporter, dCas9-TAD, and sgRNAs were co-transfected at a 2:1:1 ratio. For endogenous gene activation experiments, plasmids encoding the dCas9-TAD and sgRNAs were co-transfected at a 2:1 mass ratio. For experiments monitoring transfection efficiencies, 50 ng of a GFP expression plasmid (pZB-1) was additionally added to the 500 ng plasmid pool. Ten hours after transfection, the culture medium was replaced with fresh complete growth medium. The transfected cells were analyzed three days post-transfection for endogenous gene activation or two days post-transfection for EGFP activation. For experiments with additional puromycin selection of successfully transfected cells, the culture medium was replaced with fresh complete growth medium 24 hours after transfection. After another 24 hours, the culture medium was replaced with fresh complete growth medium supplemented

with 8 μg/mL puromycin. After selection for three days, the survived cells were collected and analyzed for BFP expression and endogenous gene activation. For electroporation of H1 cells, H1 cells were dissociated into single cells using TrypLE™ (ThermoFisher, Cat. # 12604021) at 37 °C for 5 minutes. One million cells were centrifuged at 300 g for 5 minutes, and the pellet was resuspended in 100 μL of OPTI-MEM. Subsequently, 10 μg of plasmid DNA was mixed with the cell suspension. The mixture was subjected to electroporation using the DECAY+/- mode of the BEX instrument (BEX, CUY21EDIT2) under the voltage of 150 V, the pulse duration of 5 ms, and the interval of 50 ms. During the electro-transfer phase, the voltage was set to 20 V, with pulse duration of 50 ms and interval of 50 ms. After electroporation, the cells were seeded into a matrigel-coated 12-well plate using mTeSR1 medium supplemented with 1 mM Y-27632 to promote single-cell attachment and growth. Samples were collected 72 hours post-electroporation, with medium changes performed daily.

Flow cytometry

To analyze fluorescent protein expression, cells were dissociated using 0.05% Trypsin-EDTA (Gibco, USA), resuspended in PBS with 5% FBS, and analyzed on an Attune NxT flow cytometer (Thermo Fisher Scientific, USA).

Quantitative real-time polymerase chain reaction (qRT-PCR)

Total RNA was isolated using Trizol Reagent and reverse transcribed into cDNA with the Prime Script RT Reagent Kit (Takara, Japan). qRT-PCR was performed on the Applied Biosystems QuantStudio™ 7 Pro (Thermo Fisher Scientific, USA) using SYBR Premix Ex Taq II (Takara,

Japan). qRT-PCR amplifications were performed in triplicates for each sample. The relative mRNA expression level was calculated using the $2^{-\Delta\Delta Ct}$ method. The housekeeping gene *glyceraldehyde phosphate dehydrogenase* (*GAPDH*) was used as an internal control. All qPCR primers are listed in Supplementary **Table S7**.

Yeast culture, transformation, and mCherry measurement

The *S. cerevisiae* strain CT (CEN.PK2-1c-*ura3::URA3-CYC1p-mCherry-TEF1t-TEF1p-mVenus-PGK1t*, a kind gift from Dr. Jiazhang Lian, Zhejiang University) was used as the reporter strain for mCherry activation experiments[7]. The strain was cultivated in YPD medium (10 g/liter yeast extract, 20 g/liter tryptone, 20 g/liter glucose) before transformation. Plasmid transformation of CT (1 μg of each plasmid per transformation) was carried out using the LiAc/SS carrier DNA/PEG method. After transformation, cells were incubated in SC-L medium for 2 days and then inoculated into fresh SC-L medium with an initial OD of 0.1 and cultivated for one day at 30 °C, 250 rpm. The transformed cells were then collected, resuspended in PBS, and analyzed on Attune NxT flow cytometer (Thermo Fisher Scientific, USA).

Transcriptome profiling by RNA sequencing

HEK293T cells were transfected with 4 pooled sgRNA plasmids and dCas9-NP or dCas9-VPR plasmids targeting the *HBG* locus and collected for RNA isolation three days post-transfection. Total RNA was isolated with Trizol Reagent and mRNA was enriched and fragmented for library construction. The constructed sequencing libraries were sequenced on the Illumina

HiSeq Platform with 150 bp paired-end reads, and the paired-end clean reads were aligned to the GRCh38.104 reference genome using Hisat2 (v2.0.1). Htseq (v0.6.1) was used to count the read numbers mapped to each gene. Transcripts per kilobase million (TPM) of each gene was calculated based on the length of the gene and the read count mapped to this gene. Differential expression analysis between the two groups was performed using the DESeq2 R package (v1.26.0). The resulting *P*-value of <0.05 and fold change of >2 were used to identify differentially expressed genes.

Statistical analysis

Statistical analyses were carried out with GraphPad Prism software (version 10). Error bars represent the standard error of the mean (S.E.M.) and results were presented as mean ± SEM. One-way ANOVA with Dunnett's test was used to calculate *P* values.

SUPPLEMENTARY TEXT

Using the established T Cell Class I pMHC Immunogenicity Tool from the Immune Epitope Database (Calis et al. 2013), we evaluated the immunogenicity scores of all 9-amino acid peptide sequences in human and viral TADs. A higher immunogenicity score indicates that the peptide composition is more similar to immunogenic peptides, suggesting a higher likelihood of triggering a cellular immune response. In terms of the number of fragments with positive scores, there are 169, 101, 149, and 144 fragments within NP, CN, CM, and CP fusion, respectively. These numbers are substantially smaller than VPR (256 fragments, **Table S4**). In terms of the percentage of fragments with positive scores, NP, CM, and CP all have lower percentages as compared to VPR (**Table S4**). These numbers suggest that combinatorial hTADs may exhibit less immunogenicity than viral TADs.

SUPPLEMENTARY FIGURES

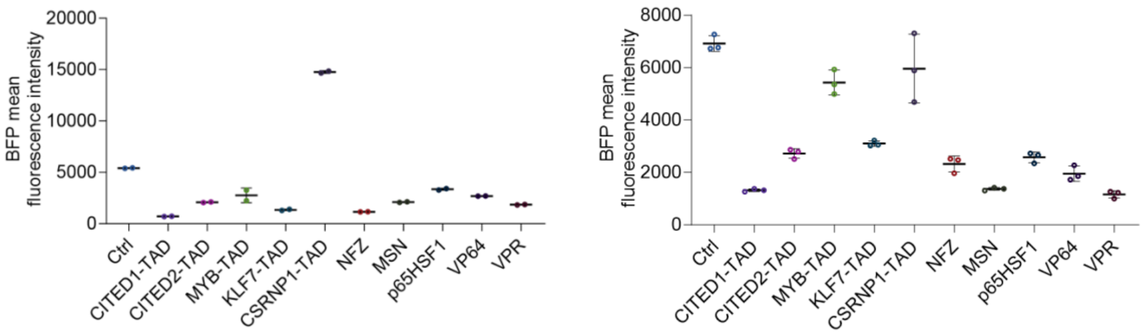

Figure S1. Expression levels of different dCas9-TADs corresponding to Fig. 1D (left panel) and Fig. 1E (right panel), respectively. Ctrl, dCas9 without TADs. Error bars represent standard error of the mean.

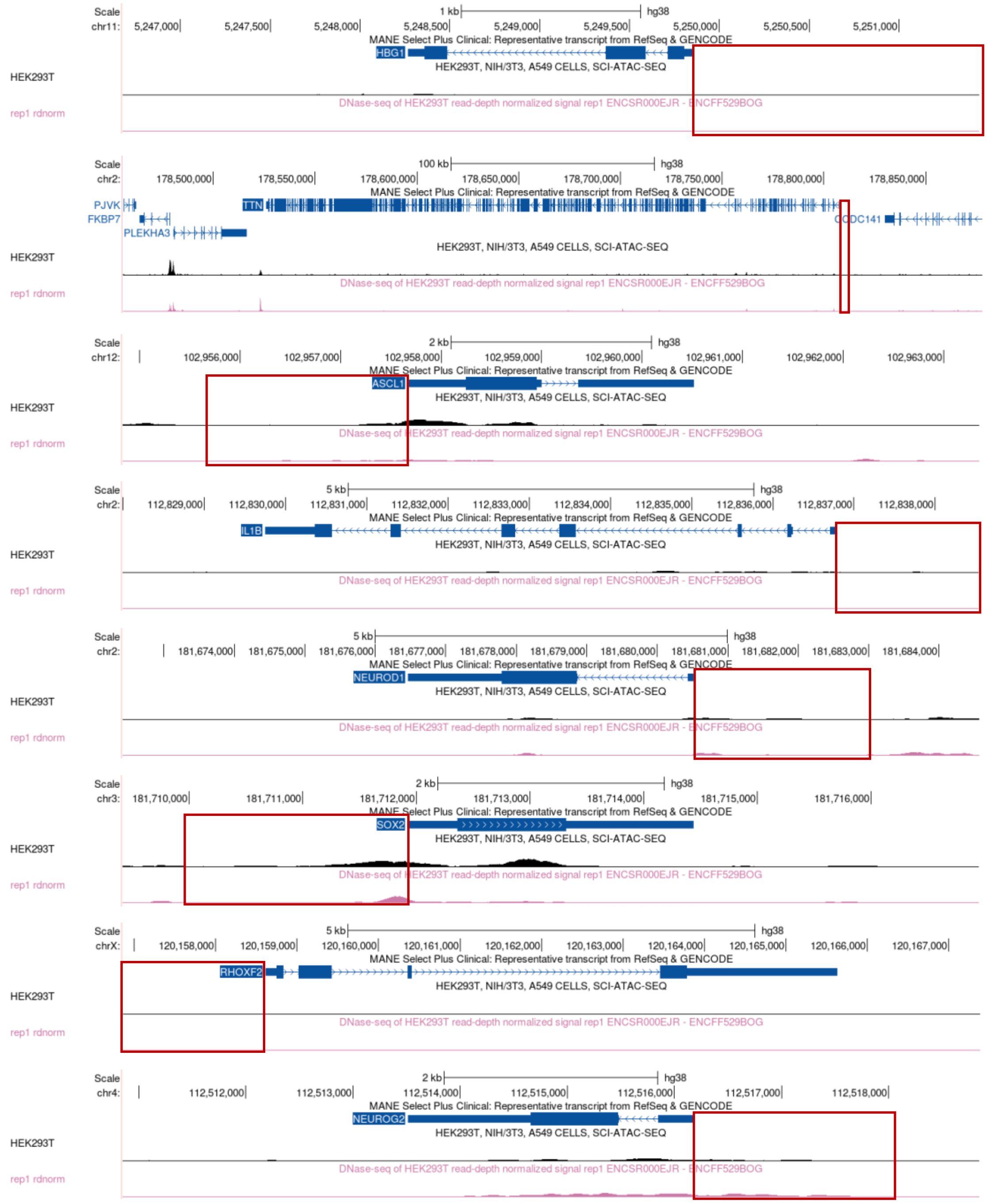

Figure S2. Chromatin accessibility of targeted genes in this study. The top track in blue indicates the representative transcript and its orientation. The middle track in black indicates the peak signal of ATAC-Seq. The bottom track in pink indicates the peak signal of DNase-Seq. The red box denotes the promoter regions of the selected genes (around a 2 kb region upstream of the transcription start site).

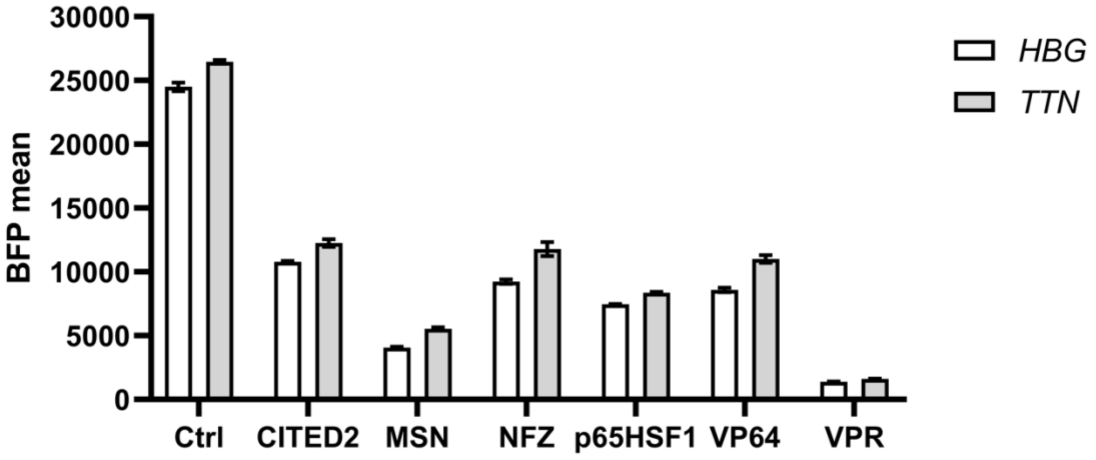

Figure S3. Expression levels of different dCas9-TADs corresponding to Fig. 1F. Ctrl, dCas9 without TADs. Error bars represent standard error of the mean.

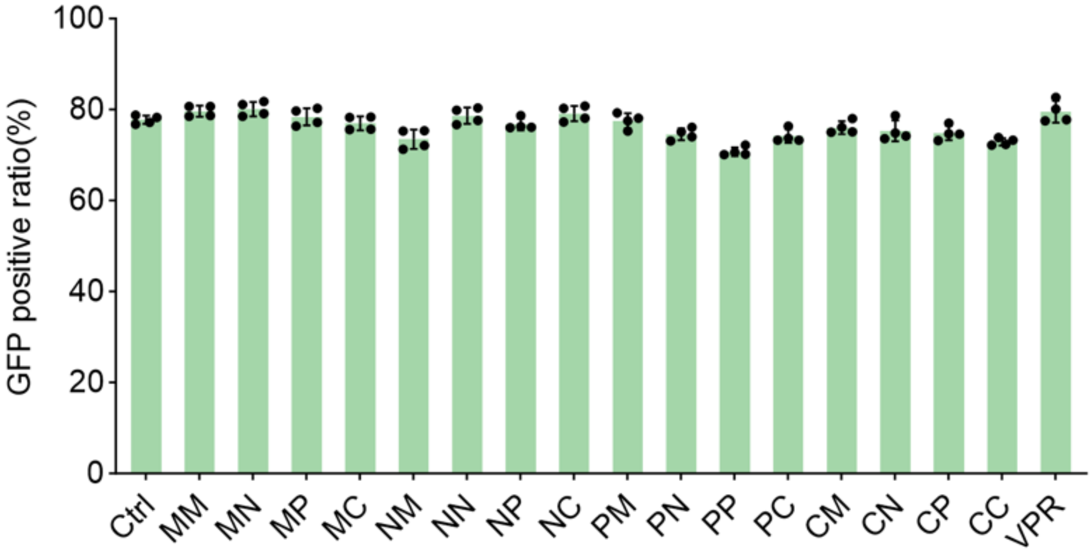

Figure S4. Transfection efficiencies of dCas9 fused pairwise combinatorial hTADs. 50 ng of a GFP expressing plasmid was spiked into each transfection reagent mix to estimate the percentage of successfully transfected cells. Ctrl, dCas9 without TADs. Error bars represent standard error of the mean.

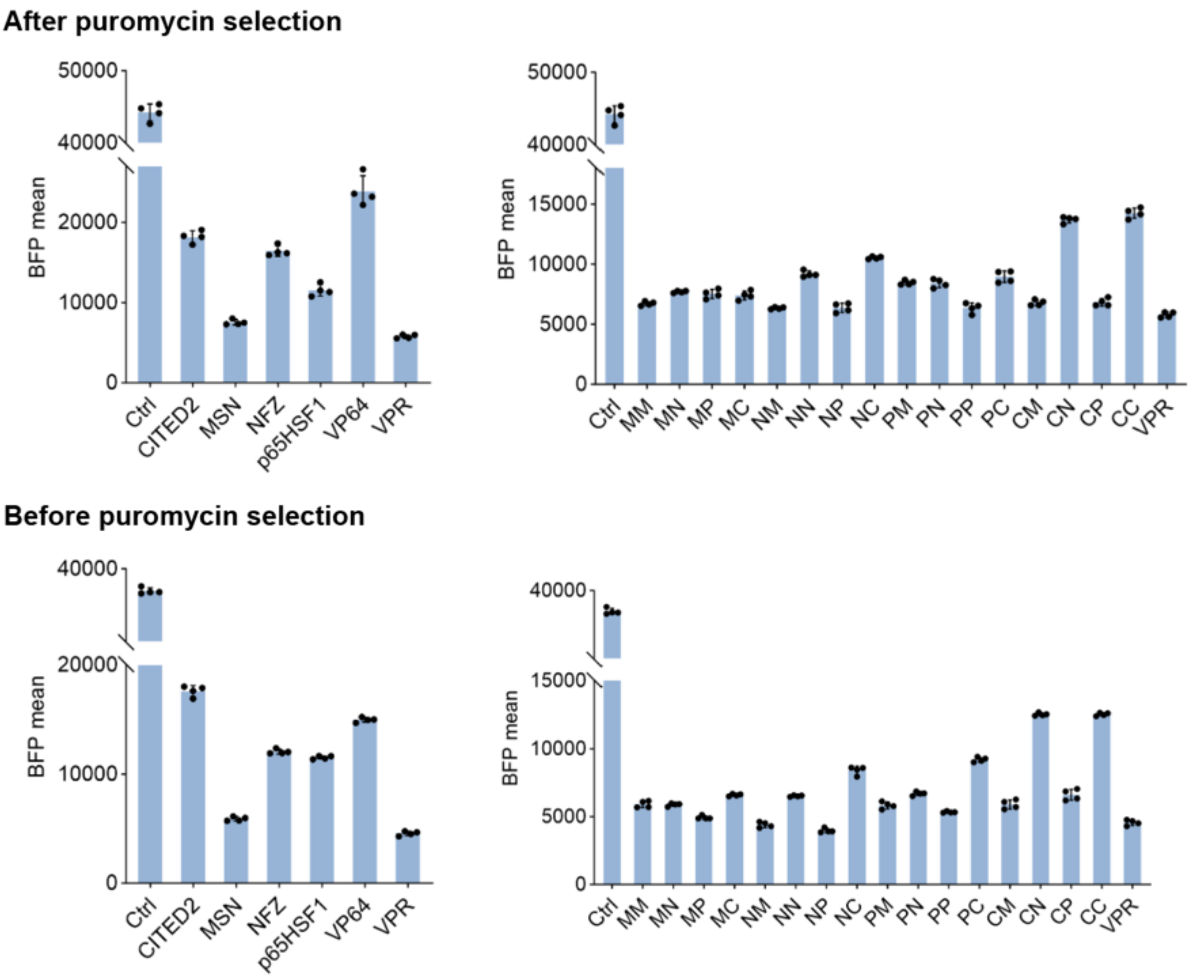

Figure S5. Expression levels of different dCas9-TADs before and after puromycin selection of successfully transfected cells. Ctrl, dCas9 without TADs. Error bars represent standard error of the mean.

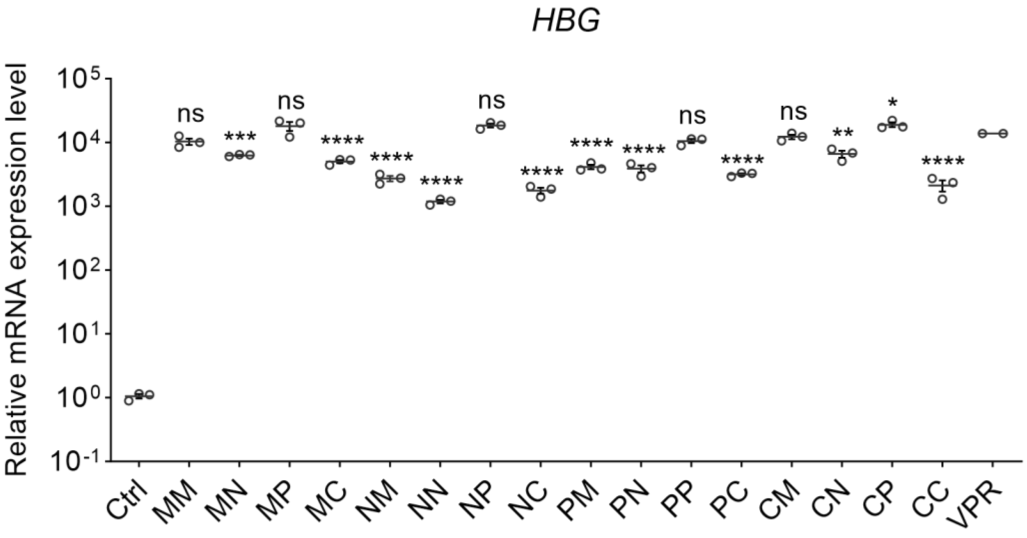

Figure S6. *HBG* activation levels of pairwise fusion hTADs with GFP-expressing plasmid spike in. Ctrl, dCas9 without TADs. Error bars represent standard error of the mean. Significance levels were calculated by one-way ANOVA followed by Dunnett's test against VPR. *, $P < 0.05$; **, $P < 0.01$; ***, $P < 0.001$; ****, $P < 0.0001$; ns, not significant.

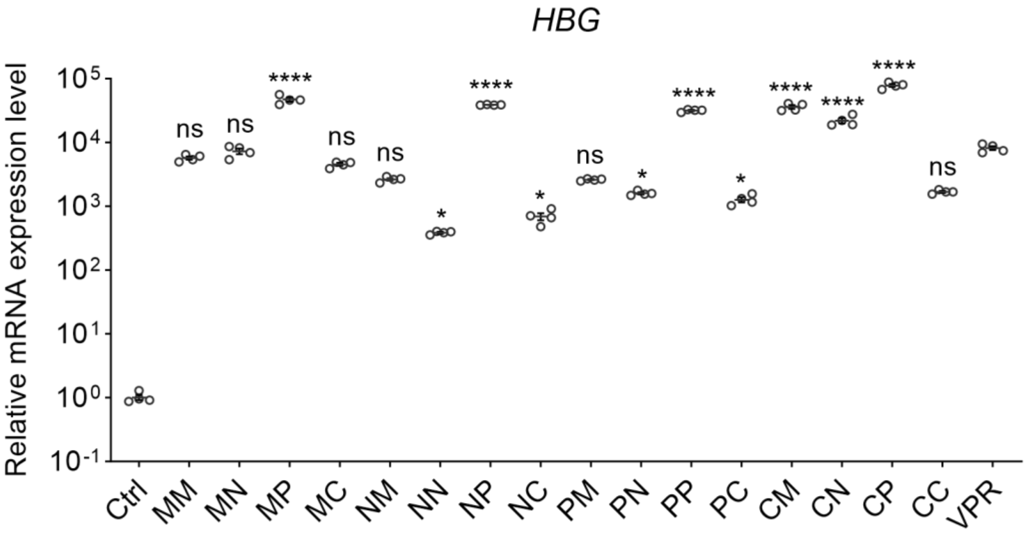

Figure S7. *HBG* activation levels of pairwise fusion hTADs after puromycin selection of successfully transfected cells. Ctrl, dCas9 without TADs. Error bars represent standard error of the mean. Significance levels were calculated by one-way ANOVA followed by Dunnett's test against VPR. *, $P < 0.05$; ****, $P < 0.0001$; ns, not significant.

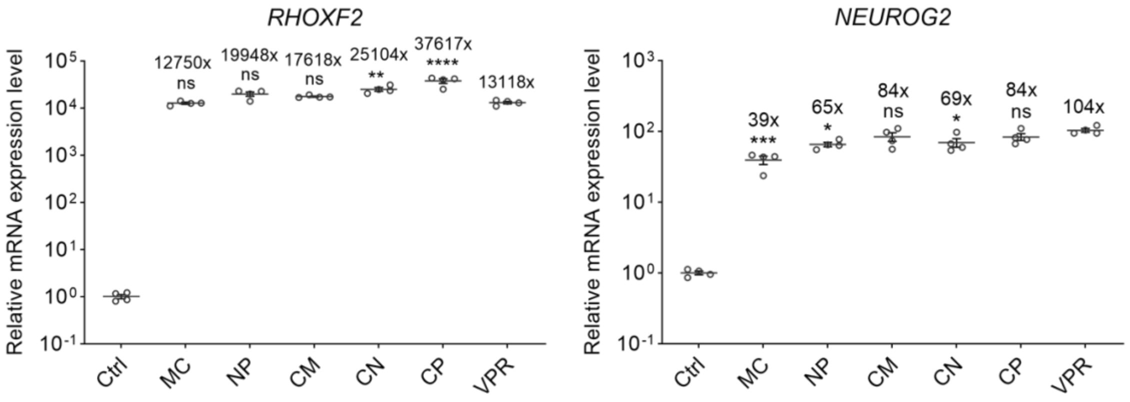

Figure S8. Relative expression levels of endogenous *RHOXF2* and *NEUROG2* after dCas9-hTADs were targeted to their respective promoters using pools of 4 sgRNAs as measured by qRT-PCR. Ctrl, dCas9 without TADs. qRT-PCR samples were collected at 72 h post-transfection. The housekeeping gene *glyceraldehyde phosphate dehydrogenase* (*GAPDH*) was used as an internal control for the normalization of qRT-PCR data. The data were graphed as mean ± S.E.M and represent four biological repeats. Significance levels were calculated by one-way ANOVA followed by Dunnett's test against VPR. *, $P < 0.05$; **, $P < 0.01$; ***, $P < 0.001$; ****, $P < 0.0001$; ns, not significant.

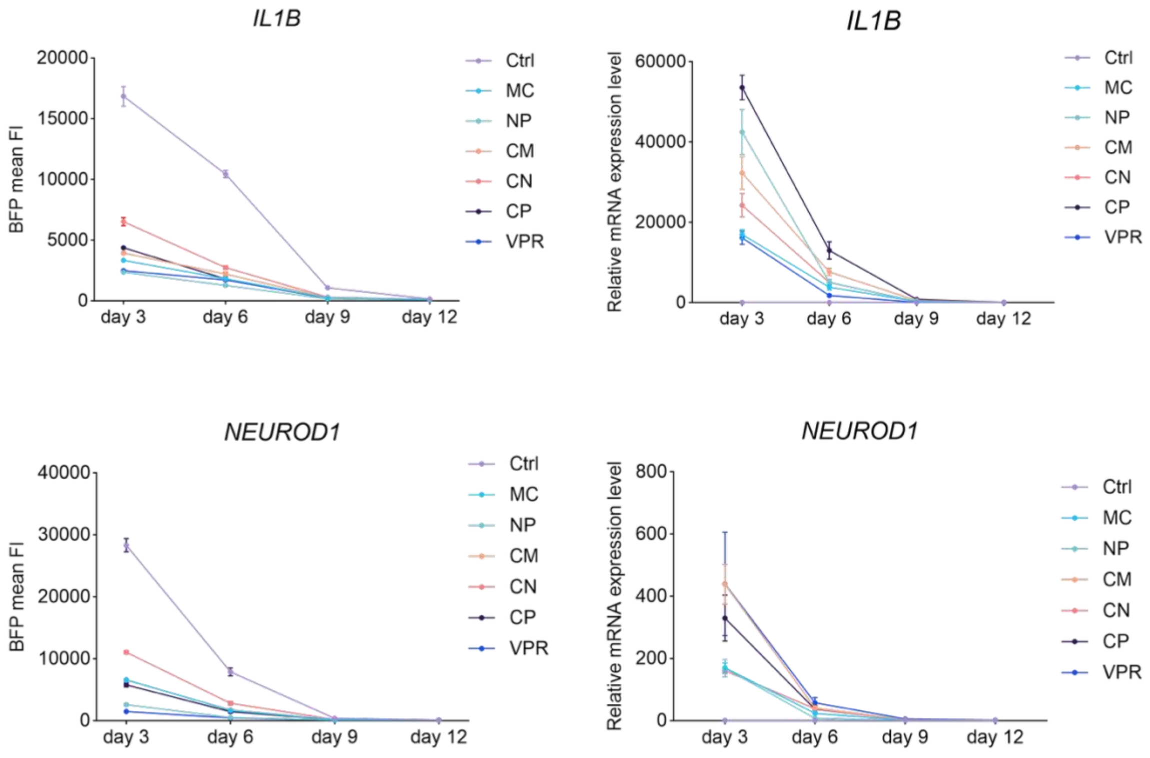

Figure S9. Expression levels (left panel) and activation levels (right panel) of different dCas9-hTADs in a longer-term experiment targeting *IL1B* and *NEUROD1* in HEK293T cells. Ctrl, dCas9 without TADs. Error bars represent standard error of the mean.

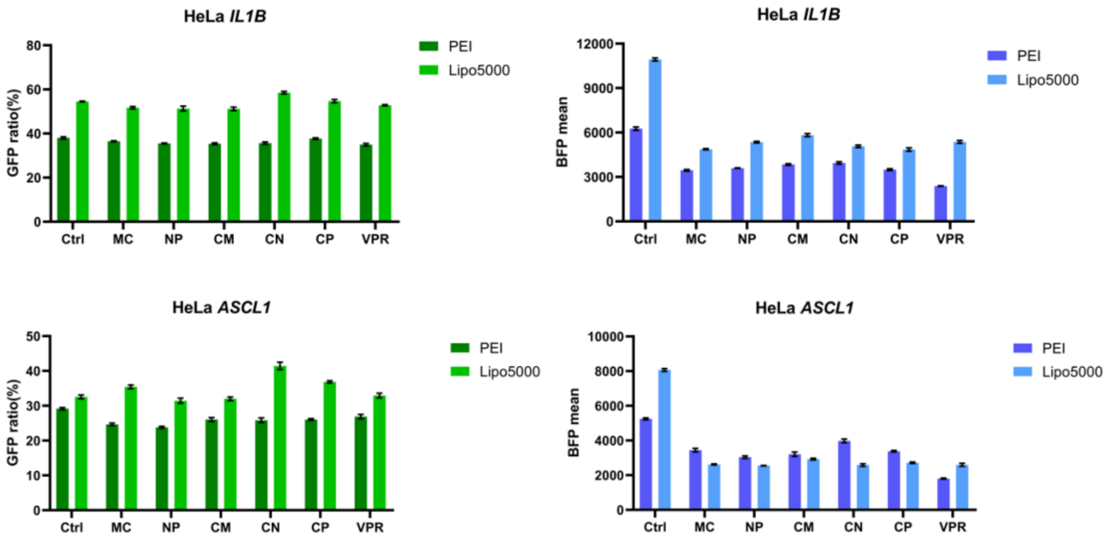

Figure S10. Transfection efficiencies (left panel) and expression levels (right panel) of different dCas9-hTADs in HeLa cells, corresponding to Fig. S9 and Fig. S10. Ctrl, dCas9 without TADs. Error bars represent standard error of the mean.

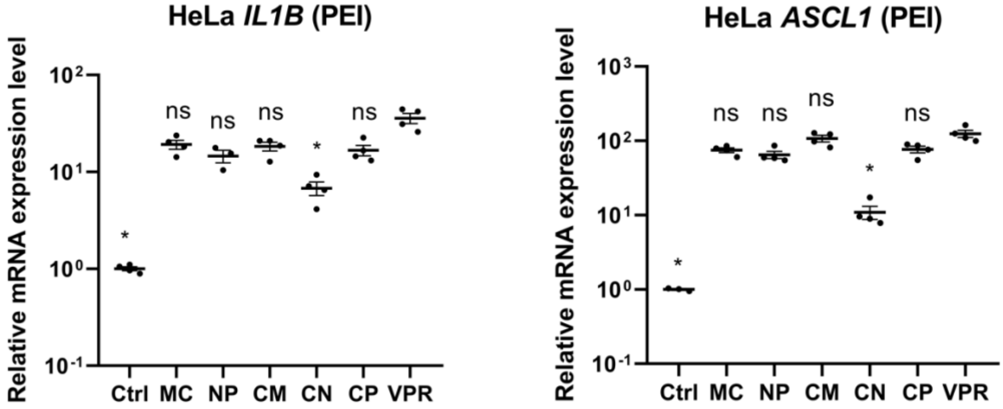

Figure S11. Relative expression levels of endogenous *IL1B* and *ASCL1* in HeLa cells using PEI as the transfection reagent. Ctrl, dCas9 without TADs. qRT-PCR samples were collected at 72 h post-transfection. The housekeeping gene *glyceraldehyde phosphate dehydrogenase* (*GAPDH*) was used as an internal control for the normalization of qRT-PCR data. The data were graphed as mean ± S.E.M and represent four biological repeats. Significance levels were calculated by one-way ANOVA followed by Dunnett's test against VPR. *, $P < 0.05$; ns, not significant.

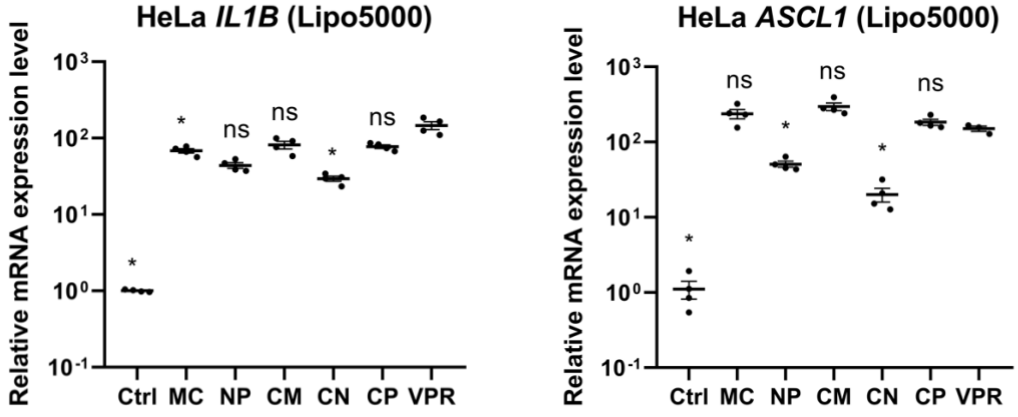

Figure S12. Relative expression levels of endogenous *IL1B* and *ASCL1* in HeLa cells using Lipofectamine 5000 as the transfection reagent. Ctrl, dCas9 without TADs. qRT-PCR samples were collected at 72 h post-transfection. The housekeeping gene *glyceraldehyde phosphate dehydrogenase* (*GAPDH*) was used as an internal control for the normalization of qRT-PCR data. The data were graphed as mean ± S.E.M and represent four biological repeats. Significance levels were calculated by one-way ANOVA followed by Dunnett's test against VPR. *, $P < 0.05$; ns, not significant.

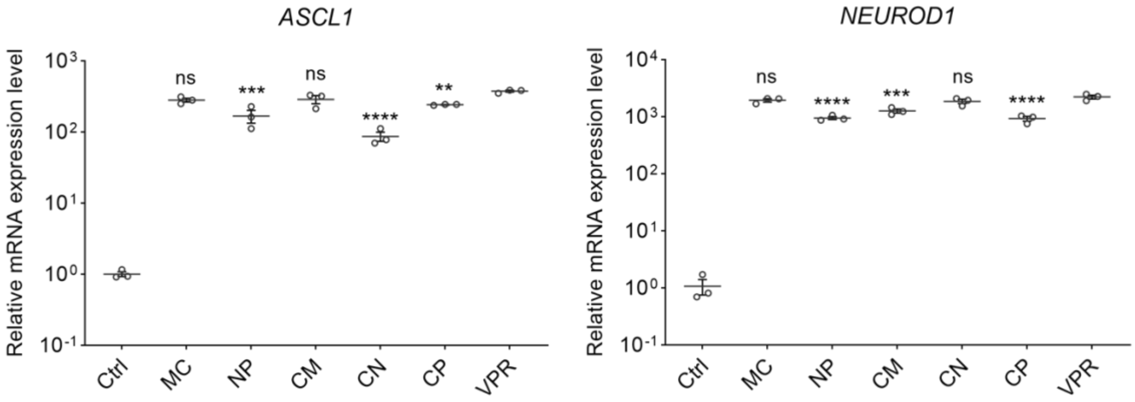

Figure S13. Relative expression levels of endogenous *ASCL1* and *NEUROD1* in hESCs. Ctrl, dCas9 without TADs. qRT-PCR samples were collected at 72 h post-electroporation. The housekeeping gene *glyceraldehyde phosphate dehydrogenase* (*GAPDH*) was used as an internal control for the normalization of qRT-PCR data. The data were graphed as mean ± S.E.M and represent three biological repeats. Significance levels were calculated by one-way ANOVA followed by Dunnett's test against VPR. **, $P < 0.01$; ***, $P < 0.001$; ****, $P < 0.0001$; ns, not significant.

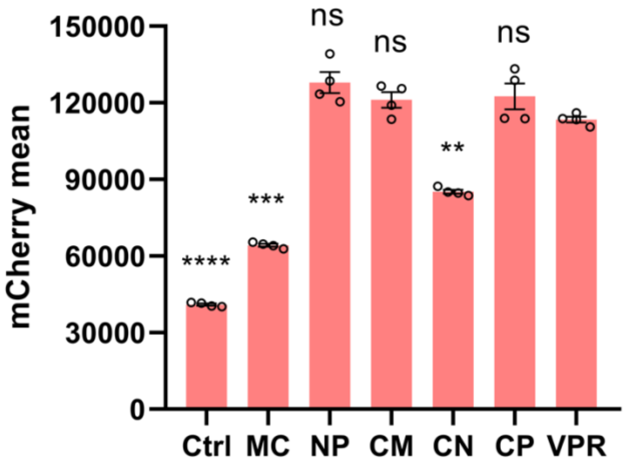

Figure S14. mCherry activation levels of dCas9-fused combinatorial hTADs in yeast. Ctrl, dCas9-VPR without sgRNA. Error bars represent standard error of the mean. Significance levels were calculated by one-way ANOVA followed by Dunnett's test against VPR. **, $P < 0.01$; ***, $P < 0.001$; ****, $P < 0.0001$; ns, not significant.

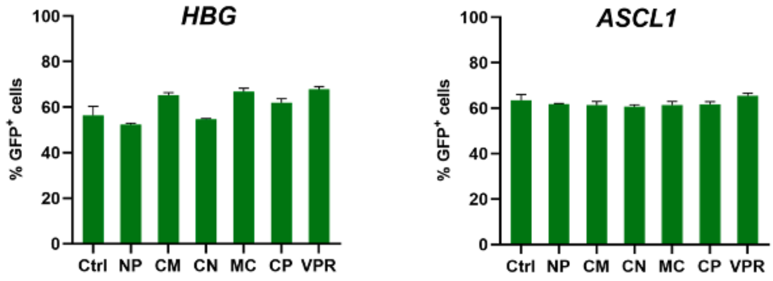

Figure S15. Transfection efficiencies of different dCasMINI-hTADs in HEK293T cells. Ctrl, dCasMINI without TADs. Error bars represent standard error of the mean.

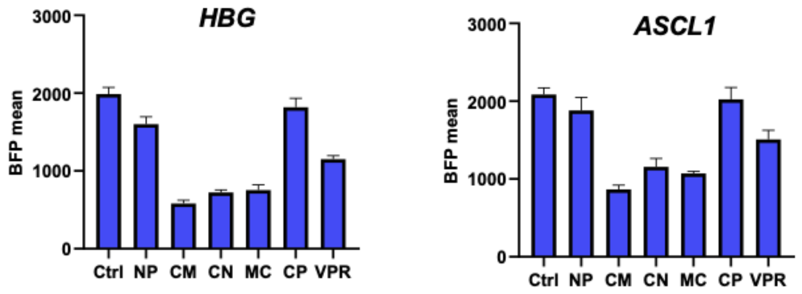

Figure S16. Expression levels of different dCasMINI-hTADs in HEK293T cells, corresponding to Figure 2I. Ctrl, dCasMINI without TADs. Error bars represent standard error of the mean.

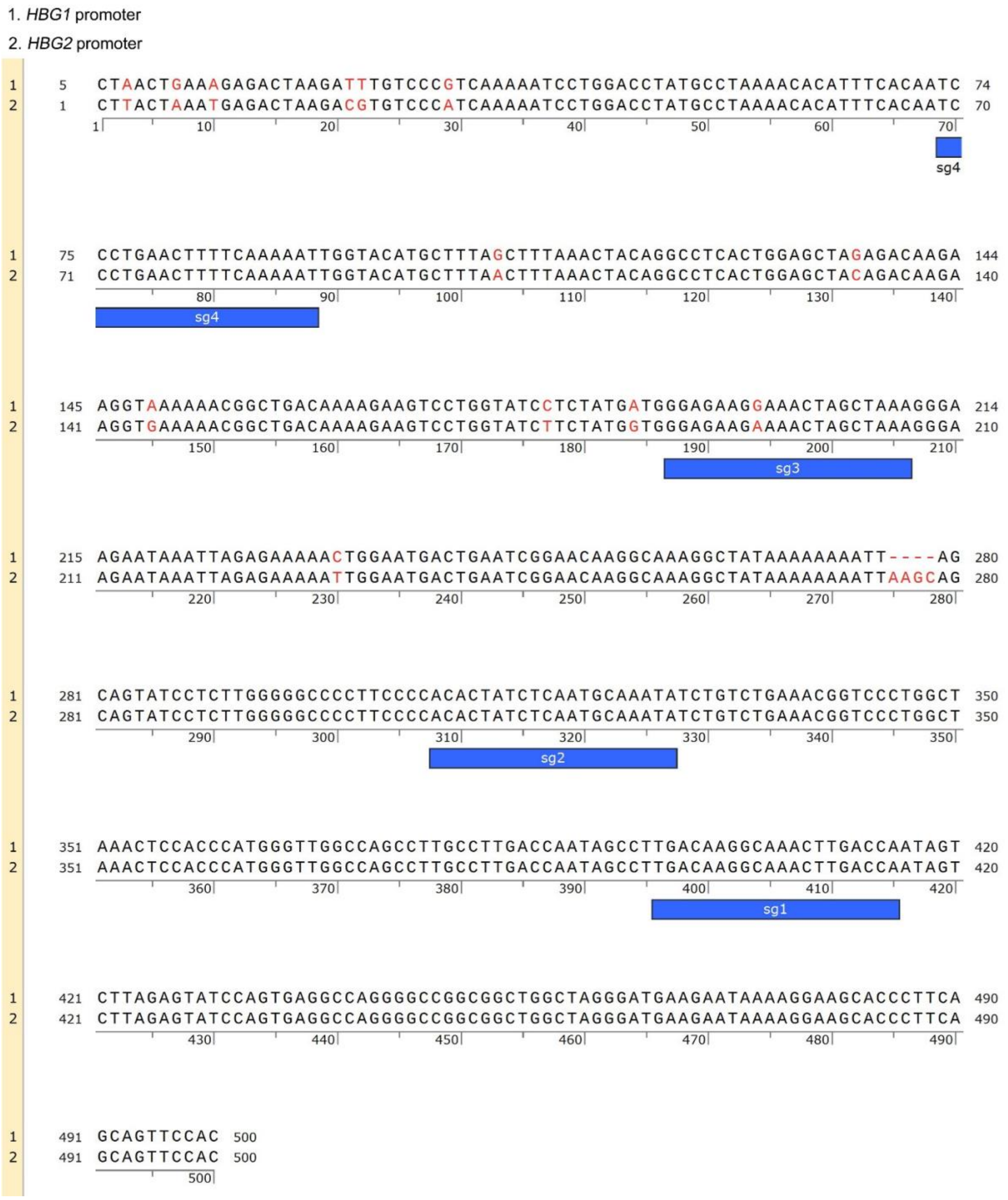

Figure S17. The promoter sequence alignment of *HBG1* and *HBG2*. The 500 bp regions upstream of the transcription initiation sites of *HBG1* and *HBG2* genes were subjected to sequence alignment. Sequence discrepancies are highlighted in red, and blue boxes denote the sgRNA sequences.

SUPPLEMENTARY TABLES

Table S1. The amino acid sequences of ATFs constructed in this study.

**dCas9-NP:** ***Streptococcus pyogenes*** **Cas9 (D10A, H840A), SV40 Nuclear Localization Sequence, NCOA3-TAD, FOXO3-TAD, ZN473-KRAB, Glysine-Serine Linker Sequence, p65, HSF1**
MDKKYSIGLAIGTNSVGWAVITDEYKVPSKKFKVLGNTDRHSIKKNLIGALLFDSG<br>ETAEATRLKRTARRRYTRRKNRICYLQEIFSNEMAKVDDSFFHRLEESFLVEEDKKH<br>ERHPIFGNIVDEVAYHEKYPTIYHLRKKLVDSTDKADLRLIYLALAHMIKFRGHFLI<br>EGDLNPDNSDVDKLFIQLVQTYNQLFEENPINASGVDAKAILSARLSKSRRLENLIA<br>QLPGEKKNGLFGNLIALSLGLTPNFKSNFDLAEDAKLQLSKDTYDDDLDNLLAQIG<br>DQYADLFLAAKNLSDAILLSDILRVNTEITKAPLSASMIKRYDEHHQDLTLLKALVR<br>QQLPEKYKEIFFDQSKNGYAGYIDGGASQEEFYKFIKPILEKMDGTEELLVKLNRED<br>LLRKQRTFDNGSIPHQIHLGELHAILRRQEDFYPFLKDNREKIEKILTFRIPYYVGPL<br>ARGNSRFAWMTRKSEETITPWNFEEVVDKGASAQSFIERMTNFDKNLPNEKVLPK<br>HSLLYEYFTVYNELTKVKYVTEGMRKPAFLSGEQKKAIVDLLFKTNRKVTVKQLK<br>EDYFKKIECFDSVEISGVEDRFNASLGTYHDLLKIIKDKDFLDNEENEDILEDIVLTL<br>TLFEDREMIEERLKTYAHLFDDKVMKQLKRRRYTGWGRLSRKLINGIRDKQSGKTI<br>LDFLKSDGFANRNFMQLIHDDSLTFKEDIQKAQVSGQGDSLHEHIANLAGSPAIKK<br>GILQTVKVVDELVKVMGRHKPENIVIEMARENQTTQKGQKNSRERMKRIEEGIKE<br>LGSQILKEHPVENTQLQNEKLYLYYLQNGRDMYVDQELDINRLSDYDVDAIVPQS<br>FLKDDSIDNKVLTRSDKNRGKSDNVPSEEVVKKMKNYWRQLLNAKLITQRKFDN<br>LTKAERGGLSELDKAGFIKRQLVETRQITKHVAQILDSRMNTKYDENDKLIREVKVI<br>TLKSKLVSDFRKDFQFYKVREINNYHHAHDAYLNAVVGTALIKKYPKLESEFVYG<br>DYKVYDVRKMIAKSEQEIGKATAKYFFYSNIMNFFKTEITLANGEIRKRPLIETNGE<br>TGEIVWDKGRDFATVRKVLSMPQVNIVKKTEVQTGGFSKESILPKRNSDKLIARKK<br>DWDPKKYGGFDSPTVAYSVLVVAKVEKGKSKKLKSVKELLGITIMERSSFEKNPID<br>FLEAKGYKEVKKDLIIKLPKYSLFELENGRKRMLASAGELQKGNELALPSKYVNFL<br>YLASHYEKLKGSPEDNEQKQLFVEQHKHYLDEIIEQISEFSKRVILADANLDKVLSA<br>YNKHRDKPIREQAENIIHLFTLTNLGAPAAFKYFDTTIDRKRYTSTKEVLDATLIHQS<br>ITGLYETRIDLSQLGGDSPKKKRKVGSEGQSDERALLDQLHTLLSNTDATGLEEIDR<br>ALGIPELVNQGQALEPKQGSGSGSHEKFPSDLDLDMFNGSLECDMESIIRSELMDA<br>DGLDFNFDSGSGSGSFVTLKDVGMDFTLGDWEQLGLEQGDTFWDTALDNCQDLF<br>LLGGGGSPSGQISNQALALAPSSAPVLAQTMVPSSAMVPLAQPPAPAPVLTPGPPQS<br>LSAPVPKSTQAGEGTLSEALLHLQFDADEDLGALLGNSTDPGVFTDLASVDNSEFQ<br>QLLNQGVSMSHSTAEPMLMEYPEAITRLVTGSQRPPDPAPTPLGTSGLPNGLSGDE<br>DFSSIADMDFSALLSQISSSGQGGGGSGFSVDTSALLDLFSPSVTVPDMSLPDLDSS<br>LASIQELLSPQEPPRPPEAENSSPDSGKQLVHYTAQPLFLLDPGSVDTGSNDLPVLFE<br>LGEGSYFSEGDGFAEDPTISLLTGSEPPKAKDPTVS
**dCas9-CP:** ***Streptococcus pyogenes*** **Cas9 (D10A, H840A), SV40 Nuclear Localization Sequence, CITED2-TAD, Glysine-Serine Linker Sequence, p65, HSF1**

MDKKYSIGLAIGTNSVGWAVITDEYKVPSKKFKVLGNTDRHSIKKNLIGALLFDSG
ETAEATRLKRTARRRYTRRKNRICYLQEIFSNEMAKVDDSFFHRLEESFLVEEDKKH
ERHPIFGNIVDEVAYHEKYPTIYHLRKKLVDSTDKADLRLIYLALAHMIKFRGHFLI
EGDLNPDNSDVDKLFIQLVQTYNQLFEENPINASGVDAKAILSARLSKSRRLENLIA
QLPGEKKNGLFGNLIALSLGLTPNFKSNFDLAEDAKLQLSKDTYDDDLDNLLAQIG
DQYADLFLAAKNLSDAILLSDILRVNTEITKAPLSASMIKRYDEHHQDLTLLKALVR
QQLPEKYKEIFFDQSKNGYAGYIDGGASQEEFYKFIKPILEKMDGTEELLVKLNRED
LLRKQRTFDNGSIPHQIHLGELHAILRRQEDFYPFLKDNREKIEKILTFRIPYYVGPL
ARGNSRFAWMTRKSEETITPWNFEEVVDKGASAQSFIERMTNFDKNLPNEKVLPK
HSLLYEYFTVYNELTKVKYVTEGMRKPAFLSGEQKKAIVDLLFKTNRKVTVKQLK
EDYFKKIECFDSVEISGVEDRFNASLGTYHDLLKIIKDKDFLDNEENEDILEDIVLTL
TLFEDREMIEERLKTYAHLFDDKVMKQLKRRRYTGWGRLSRKLINGIRDKQSGKTI
LDFLKSDGFANRNFMQLIHDDSLTFKEDIQKAQVSGQGDSLHEHIANLAGSPAIKK
GILQTVKVVDELVKVMGRHKPENIVIEMARENQTTQKGQKNSRERMKRIEEGIKE
LGSQILKEHPVENTQLQNEKLYLYYLQNGRDMYVDQELDINRLSDYDVDAIVPQS
FLKDDSIDNKVLTRSDKNRGKSDNVPSEEVVKKMKNYWRQLLNAKLITQRKFDN
LTKAERGGLSELDKAGFIKRQLVETRQITKHVAQILDSRMNTKYDENDKLIREVKVI
TLKSKLVSDFRKDFQFYKVREINNYHHAHDAYLNAVVGTALIKKYPKLESEFVYG
DYKVYDVRKMIAKSEQEIGKATAKYFFYSNIMNFFKTEITLANGEIRKRPLIETNGE
TGEIVWDKGRDFATVRKVLSMPQVNIVKKTEVQTGGFSKESILPKRNSDKLIARKK
DWDPKKYGGFDSPTVAYSVLVVAKVEKGKSKKLKSVKELLGITIMERSSFEKNPID
FLEAKGYKEVKKDLIIKLPKYSLFELENGRKRMLASAGELQKGNELALPSKYVNFL
YLASHYEKLKGSPEDNEQKQLFVEQHKHYLDEIIEQISEFSKRVILADANLDKVLSA
YNKHRDKPIREQAENIIHLFTLTNLGAPAAFKYFDTTIDRKRYTSTKEVLDATLIHQS
ITGLYETRIDLSQLGGDSPKKKRKVGSAMLPPNVIDTDFIDEEVLMSLVIEMGLDRI
KELPELWLGQNEFDFMTDFVCKQQPSRVSCGGGGSPSGQISNQALALAPSSAPVLA
QTMVPSSAMVPLAQPPAPAPVLTPGPPQSLSAPVPKSTQAGEGTLSEALLHLQFDA
DEDLGALLGNSTDPGVFTDLASVDNSEFQQLLNQGVSMSHSTAEPMLMEYPEAIT
RLVTGSQRPPDPAPTPLGTSGLPNGLSGDEDFSSIADMDFSALLSQISSSGQGGGGS
GFSVDTSALLDLFSPSVTVPDMSLPDLDSSLASIQELLSPQEPPRPPEAENSSPDSGK
QLVHYTAQPLFLLDPGSVDTGSNDLPVLFELGEGSYFSEGDGFAEDPTISLLTGSEPP
KAKDPTVS

dCas9-CN: ***Streptococcus pyogenes*** **Cas9 (D10A, H840A), SV40 Nuclear Localization Sequence, CITED2-TAD, Glysine-Serine Linker Sequence, NCOA3-TAD, FOXO3-TAD, ZN473-KRAB**

MDKKYSIGLAIGTNSVGWAVITDEYKVPSKKFKVLGNTDRHSIKKNLIGALLFDSG
ETAEATRLKRTARRRYTRRKNRICYLQEIFSNEMAKVDDSFFHRLEESFLVEEDKKH
ERHPIFGNIVDEVAYHEKYPTIYHLRKKLVDSTDKADLRLIYLALAHMIKFRGHFLI
EGDLNPDNSDVDKLFIQLVQTYNQLFEENPINASGVDAKAILSARLSKSRRLENLIA
QLPGEKKNGLFGNLIALSLGLTPNFKSNFDLAEDAKLQLSKDTYDDDLDNLLAQIG
DQYADLFLAAKNLSDAILLSDILRVNTEITKAPLSASMIKRYDEHHQDLTLLKALVR
QQLPEKYKEIFFDQSKNGYAGYIDGGASQEEFYKFIKPILEKMDGTEELLVKLNRED
LLRKQRTFDNGSIPHQIHLGELHAILRRQEDFYPFLKDNREKIEKILTFRIPYYVGPL
ARGNSRFAWMTRKSEETITPWNFEEVVDKGASAQSFIERMTNFDKNLPNEKVLPK

HSLLYEYFTVYNELTKVKYVTEGMRKPAFLSGEQKKAIVDLLFKTNRKVTVKQLK EDYFKKIECFDSVEISGVEDRFNASLGTYHDLLKIIKDKDFLDNEENEDILEDIVLTL TLFEDREMIEERLKTYAHLFDDKVMKQLKRRRYTGWGRLSRKLINGIRDKQSGKTI LDFLKSDGFANRNFMQLIHDDSLTFKEDIQKAQVSGQGDSLHEHIANLAGSPAIKK GILQTVKVVDELVKVMGRHKPENIVIEMARENQTTQKGQKNSRERMKRIEEGIKE LGSQILKEHPVENTQLQNEKLYLYYLQNGRDMYVDQELDINRLSDYDVDAIVPQS FLKDDSIDNKVLTRSDKNRGKSDNVPSEEVVKKMKNYWRQLLNAKLITQRKFDN LTKAERGGLSELDKAGFIKRQLVETRQITKHVAQILDSRMNTKYDENDKLIREVKVI TLKSKLVSDFRKDFQFYKVREINNYHHAHDAYLNAVVGTALIKKYPKLESEFVYG DYKVYDVRKMIAKSEQEIGKATAKYFFYSNIMNFFKTEITLANGEIRKRPLIETNGE TGEIVWDKGRDFATVRKVLSMPQVNIVKKTEVQTGGFSKESILPKRNSDKLIARKK DWDPKKYGGFDSPTVAYSVLVVAKVEKGKSKKLKSVKELLGITIMERSSFEKNPID FLEAKGYKEVKKDLIIKLPKYSLFELENGRKRMLASAGELQKGNELALPSKYVNFL YLASHYEKLKGSPEDNEQKQLFVEQHKHYLDEIIEQISEFSKRVILADANLDKVLSA YNKHRDKPIREQAENIIHLFTLTNLGAPAAFKYFDTTIDRKRYTSTKEVLDATLIHQS ITGLYETRIDLSQLGGDSPKKKRKVGSAMLPPNVIDTDFIDEEVLMSLVIEMGLDRI KELPELWLGQNEFDFMTDFVCKQQPSRVSCGGGGSEGQSDERALLDQLHTLLSNT DATGLEEIDRALGIPELVNQGQALEPKQGSGSGSHEKFPSDLDLDMFNGSLECDME SIIRSELMDADGLDFNFDSGSGSGSFVTLKDVGMDFTLGDWEQLGLEQGDTFWDT ALDNCQDLFLL
**dCas9-CM:** ***Streptococcus pyogenes*** **Cas9 (D10A, H840A), SV40 Nuclear Localization Sequence, CITED2-TAD, Glysine-Serine Linker Sequence, MRTF-A-TAD, STAT1-TAD, Neh4-Neh5-TAD**
MDKKYSIGLAIGTNSVGWAVITDEYKVPSKKFKVLGNTDRHSIKKNLIGALLFDSG ETAEATRLKRTARRRYTRRKNRICYLQEIFSNEMAKVDDSFFHRLEESFLVEEDKKH ERHPIFGNIVDEVAYHEKYPTIYHLRKKLVDSTDKADLRLIYLALAHMIKFRGHFLI EGDLNPDNSDVDKLFIQLVQTYNQLFEENPINASGVDAKAILSARLSKSRRLENLIA QLPGEKKNGLFGNLIALSLGLTPNFKSNFDLAEDAKLQLSKDTYDDDLDNLLAQIG DQYADLFLAAKNLSDAILLSDILRVNTEITKAPLSASMIKRYDEHHQDLTLLKALVR QQLPEKYKEIFFDQSKNGYAGYIDGGASQEEFYKFIKPILEKMDGTEELLVKLNRED LLRKQRTFDNGSIPHQIHLGELHAILRRQEDFYPFLKDNREKIEKILTFRIPYYVGPL ARGNSRFAWMTRKSEETITPWNFEEVVDKGASAQSFIERMTNFDKNLPNEKVLPK HSLLYEYFTVYNELTKVKYVTEGMRKPAFLSGEQKKAIVDLLFKTNRKVTVKQLK EDYFKKIECFDSVEISGVEDRFNASLGTYHDLLKIIKDKDFLDNEENEDILEDIVLTL TLFEDREMIEERLKTYAHLFDDKVMKQLKRRRYTGWGRLSRKLINGIRDKQSGKTI LDFLKSDGFANRNFMQLIHDDSLTFKEDIQKAQVSGQGDSLHEHIANLAGSPAIKK GILQTVKVVDELVKVMGRHKPENIVIEMARENQTTQKGQKNSRERMKRIEEGIKE LGSQILKEHPVENTQLQNEKLYLYYLQNGRDMYVDQELDINRLSDYDVDAIVPQS FLKDDSIDNKVLTRSDKNRGKSDNVPSEEVVKKMKNYWRQLLNAKLITQRKFDN LTKAERGGLSELDKAGFIKRQLVETRQITKHVAQILDSRMNTKYDENDKLIREVKVI TLKSKLVSDFRKDFQFYKVREINNYHHAHDAYLNAVVGTALIKKYPKLESEFVYG DYKVYDVRKMIAKSEQEIGKATAKYFFYSNIMNFFKTEITLANGEIRKRPLIETNGE TGEIVWDKGRDFATVRKVLSMPQVNIVKKTEVQTGGFSKESILPKRNSDKLIARKK DWDPKKYGGFDSPTVAYSVLVVAKVEKGKSKKLKSVKELLGITIMERSSFEKNPID

FLEAKGYKEVKKDLIIKLPKYSLFELENGRKRMLASAGELQKGNELALPSKYVNFL
YLASHYEKLKGSPEDNEQKQLFVEQHKHYLDEIIEQISEFSKRVILADANLDKVLSA
YNKHRDKPIREQAENIIHLFTLTNLGAPAAFKYFDTTIDRKRYTSTKEVLDATLIHQS
ITGLYETRIDLSQLGGDSPKKKRKVGSAMLPPNVIDTDFIDEEVLMSLVIEMGLDRI
KELPELWLGQNEFDFMTDFVCKQQPSRVSCGGGGSSSSQQMDDLFDILIQSGEISA
DFKEPPSLPGKEKPSPKTVCGSPLAAQPSPSAELPQAAPPPPGSPSLPGRLEDFLESS
TGLPLLTSGHDGPEPLSLIDDLHSQMLSSTAILDHPPSPMDTSELHFVPEPSSTMGLD
LADGHLDSMDWLELSSGGPVLSLAPLSTTAPSLFSTDFLDGHDLQLHWDSGSSEV
HPSRLQTTDNLLPMSPEEFDEVSRIVGSVEFDSASSDALYFDDCMQLLAQTFPFVD
DNESGGGSGGSGSSQDIEQVWEELLSIPELQCLNIENDKLVE

dCas9-MC: ***Streptococcus pyogenes*** **Cas9 (D10A, H840A)**, **SV40 Nuclear Localization Sequence**, **MRTF-A-TAD**, **STAT1-TAD**, **Neh4-Neh5-TAD**, **Glysine-Serine Linker Sequence**, **CITED2-TAD**

MDKKYSIGLAIGTNSVGWAVITDEYKVPSKKFKVLGNTDRHSIKKNLIGALLFDSG
ETAEATRLKRTARRRYTRRKNRICYLQEIFSNEMAKVDDSFFHRLEESFLVEEDKKH
ERHPIFGNIVDEVAYHEKYPTIYHLRKKLVDSTDKADLRLIYLALAHMIKFRGHFLI
EGDLNPDNSDVDKLFIQLVQTYNQLFEENPINASGVDAKAILSARLSKSRRLENLIA
QLPGEKKNGLFGNLIALSLGLTPNFKSNFDLAEDAKLQLSKDTYDDDLDNLLAQIG
DQYADLFLAAKNLSDAILLSDILRVNTEITKAPLSASMIKRYDEHHQDLTLLKALVR
QQLPEKYKEIFFDQSKNGYAGYIDGGASQEEFYKFIKPILEKMDGTEELLVKLNRED
LLRKQRTFDNGSIPHQIHLGELHAILRRQEDFYPFLKDNREKIEKILTFRIPYYVGPL
ARGNSRFAWMTRKSEETITPWNFEEVVDKGASAQSFIERMTNFDKNLPNEKVLPK
HSLLYEYFTVYNELTKVKYVTEGMRKPAFLSGEQKKAIVDLLFKTNRKVTVKQLK
EDYFKKIECFDSVEISGVEDRFNASLGTYHDLLKIIKDKDFLDNEENEDILEDIVLTL
TLFEDREMIEERLKTYAHLFDDKVMKQLKRRRYTGWGRLSRKLINGIRDKQSGKTI
LDFLKSDGFANRNFMQLIHDDSLTFKEDIQKAQVSGQGDSLHEHIANLAGSPAIKK
GILQTVKVVDELVKVMGRHKPENIVIEMARENQTTQKGQKNSRERMKRIEEGIKE
LGSQILKEHPVENTQLQNEKLYLYYLQNGRDMYVDQELDINRLSDYDVDAIVPQS
FLKDDSIDNKVLTRSDKNRGKSDNVPSEEVVKKMKNYWRQLLNAKLITQRKFDN
LTKAERGGLSELDKAGFIKRQLVETRQITKHVAQILDSRMNTKYDENDKLIREVKVI
TLKSKLVSDFRKDFQFYKVREINNYHHAHDAYLNAVVGTALIKKYPKLESEFVYG
DYKVYDVRKMIAKSEQEIGKATAKYFFYSNIMNFFKTEITLANGEIRKRPLIETNGE
TGEIVWDKGRDFATVRKVLSMPQVNIVKKTEVQTGGFSKESILPKRNSDKLIARKK
DWDPKKYGGFDSPTVAYSVLVVAKVEKGKSKKLKSVKELLGITIMERSSFEKNPID
FLEAKGYKEVKKDLIIKLPKYSLFELENGRKRMLASAGELQKGNELALPSKYVNFL
YLASHYEKLKGSPEDNEQKQLFVEQHKHYLDEIIEQISEFSKRVILADANLDKVLSA
YNKHRDKPIREQAENIIHLFTLTNLGAPAAFKYFDTTIDRKRYTSTKEVLDATLIHQS
ITGLYETRIDLSQLGGDSPKKKRKVGSSSSQQMDDLFDILIQSGEISADFKEPPSLPG
KEKPSPKTVCGSPLAAQPSPSAELPQAAPPPPGSPSLPGRLEDFLESSTGLPLLTSGH
DGPEPLSLIDDLHSQMLSSTAILDHPPSPMDTSELHFVPEPSSTMGLDLADGHLDSM
DWLELSSGGPVLSLAPLSTTAPSLFSTDFLDGHDLQLHWDSGSSEVHPSRLQTTDN
LLPMSPEEFDEVSRIVGSVEFDSASSDALYFDDCMQLLAQTFPFVDDNESGGGSGG
SGSSQDIEQVWEELLSIPELQCLNIENDKLVEGGGGSAMLPPNVIDTDFIDEEVLMS
LVIEMGLDRIKELPELWLGQNEFDFMTDFVCKQQPSRVSC

dCas9-VPR: ***Streptococcus pyogenes*** **Cas9 (D10A, H840A), SV40 Nuclear Localization Sequence, VPR**

MDKKYSIGLAIGTNSVGWAVITDEYKVPSKKFKVLGNTDRHSIKKNLIGALLFDSG
ETAEATRLKRTARRRYTRRKNRICYLQEIFSNEMAKVDDSFFHRLEESFLVEEDKKH
ERHPIFGNIVDEVAYHEKYPTIYHLRKKLVDSTDKADLRLIYLALAHMIKFRGHFLI
EGDLNPDNSDVDKLFIQLVQTYNQLFEENPINASGVDAKAILSARLSKSRRLENLIA
QLPGEKKNGLFGNLIALSLGLTPNFKSNFDLAEDAKLQLSKDTYDDDLDNLLAQIG
DQYADLFLAAKNLSDAILLSDILRVNTEITKAPLSASMIKRYDEHHQDLTLLKALVR
QQLPEKYKEIFFDQSKNGYAGYIDGGASQEEFYKFIKPILEKMDGTEELLVKLNRED
LLRKQRTFDNGSIPHQIHLGELHAILRRQEDFYPFLKDNREKIEKILTFRIPYYVGPL
ARGNSRFAWMTRKSEETITPWNFEEVVDKGASAQSFIERMTNFDKNLPNEKVLPK
HSLLYEYFTVYNELTKVKYVTEGMRKPAFLSGEQKKAIVDLLFKTNRKVTVKQLK
EDYFKKIECFDSVEISGVEDRFNASLGTYHDLLKIIKDKDFLDNEENEDILEDIVLTL
TLFEDREMIEERLKTYAHLFDDKVMKQLKRRRYTGWGRLSRKLINGIRDKQSGKTI
LDFLKSDGFANRNFMQLIHDDSLTFKEDIQKAQVSGQGDSLHEHIANLAGSPAIKK
GILQTVKVVDELVKVMGRHKPENIVIEMARENQTTQKGQKNSRERMKRIEEGIKE
LGSQILKEHPVENTQLQNEKLYLYYLQNGRDMYVDQELDINRLSDYDVDAIVPQS
FLKDDSIDNKVLTRSDKNRGKSDNVPSEEVVKKMKNYWRQLLNAKLITQRKFDN
LTKAERGGLSELDKAGFIKRQLVETRQITKHVAQILDSRMNTKYDENDKLIREVKVI
TLKSKLVSDFRKDFQFYKVREINNYHHAHDAYLNAVVGTALIKKYPKLESEFVYG
DYKVYDVRKMIAKSEQEIGKATAKYFFYSNIMNFFKTEITLANGEIRKRPLIETNGE
TGEIVWDKGRDFATVRKVLSMPQVNIVKKTEVQTGGFSKESILPKRNSDKLIARKK
DWDPKKYGGFDSPTVAYSVLVVAKVEKGKSKKLKSVKELLGITIMERSSFEKNPID
FLEAKGYKEVKKDLIIKLPKYSLFELENGRKRMLASAGELQKGNELALPSKYVNFL
YLASHYEKLKGSPEDNEQKQLFVEQHKHYLDEIIEQISEFSKRVILADANLDKVLSA
YNKHRDKPIREQAENIIHLFTLTNLGAPAAFKYFDTTIDRKRYTSTKEVLDATLIHQS
ITGLYETRIDLSQLGGDSPKKKRKVGSATEFEASGSGRADALDDFDLDMLGSDALD
DFDLDMLGSDALDDFDLDMLGSDALDDFDLDMLINSRSSGSPKKKRKVGSQYLP
DTDDRHRIEEKRKRTYETFKSIMKKSPFSGPTDPRPPPRRIAVPSRSSASVPKPAPQP
YPFTSSLSTINYDEFPTMVFPSGQISQASALAPAPPQVLPQAPAPAPAPAMVSALAQA
PAPVPVLAPGPPQAVAPPAPKPTQAGEGTLSEALLQLQFDDEDLGALLGNSTDPAVF
TDLASVDNSEFQQLLNQGIPVAPHTTEPMLMEYPEAITRLVTGAQRPPDPAPAPLGA
PGLPNGLLSGDEDFSSIADMDFSALLGSGSGSRDSREGMFLPKPEAGSAISDVFEGR
EVCQPKRIRPFHPPGSPWANRPLPASLAPTPTGPVHEPVGSLTPAPVPQPLDPAPAVT
PEASHLLEDPDEETSQAVKALREMADTVIPQKEEAAICGQMDLSHPPPRGHLDELT
TTLESMTEDLNLDSPLTPELNEILDTFLNDECLLHAMHISTGLSIFDTSLF

dCasMINI-NP: Cas12f (D326A/D510A/D143R/T147R/K330R/E528R), SV40 Nuclear Localization Sequence, NCOA3-TAD, FOXO3-TAD, ZN473-KRAB, Glysine-Serine Linker Sequence, p65, HSF1

MAKNTITKTLKLRIVRPYNSAEVEKIVADEKNNREKIALEKNKDKVKEACSKHLK
VAAYCTTQVERNACLFCKARKLDDKFYQKLRGQFPDAVFWQEISEIFRQLQKQAA
EIYNQSLIELYYEIFIKGKGIANASSVEHYLSRVCYRRAAELFKNAAIASGLRSKIKS
NFRLKELKNMKSGLPTTKSDNFPIPLVKQKGGQYTGFEISNHNSDFIIKIPFGRWQV
KKEIDKYRPWEKFDFEQVQKSPKPISLLLSTQRRKRNKGWSKDEGTEAEIKKVMN

GDYQTSYIEVKRGSKICEKSAWMLNLSIDVPKIDKGVDPSIIGGIAVGVRSPLVCAIN NAFSRYSISDNDLFHFNKKMFARRRILLKKNRHKRAGHGAKNKLKPITILTEKSERF RKKLIERWACEIADFFIKNKVGTVQMENLESMKRKEDSYFNIRLRGFWPYAEMQN KIEFKLKQYGIEIRKVAPNNTSKTCSKCGHLNNYFNFEYRKKNKFPHFKCEKCNFK ENAAYNAALNISNPKLKSTKERPAPKKKRKVGSATEFSREGQSDERALLDQLHTLL SNTDATGLEEIDRALGIPELVNQGQALEPKQGSGSGSHEKFPSDLDLDMFNGSLEC DMESIIRSELMDADGLDFNFDSGSGSGSFVTLKDVGMDFTLGDWEQLGLEQGDTF WDTALDNCQDLFLLGGGGSPSGQISNQALALAPSSAPVLAQTMVPSSAMVPLAQP PAPAPVLTPGPPQSLSAPVPKSTQAGEGTLSEALLHLQFDADEDLGALLGNSTDPGV FTDLASVDNSEFQQLLNQGVSMSHSTAEPMLMEYPEAITRLVTGSQRPPDPAPTPL GTSGLPNGLSGDEDFSSIADMDFSALLSQISSSGQGGGGSGFSVDTSALLDLFSPSV TVPDMSLPDLDSSLASIQELLSPQEPPRPPEAENSSPDSGKQLVHYTAQPLFLLDPGS VDTGSNDLPVLFELGEGSYFSEGDGFAEDPTISLLTGSEPPKAKDPTVS
**dCasMINI-CP: Cas12f (D326A/D510A/D143R/T147R/K330R/E528R), SV40 Nuclear Localization Sequence, CITED2-TAD, Glysine-Serine Linker Sequence, p65, HSF1**
MAKNTITKTLKLRIVRPYNSAEVEKIVADEKNNREKIALEKNKDKVKEACSKHLK VAAYCTTQVERNACLFCKARKLDDKFYQKLRGQFPDAVFWQEISEIFRQLQKQAA EIYNQSLIELYYEIFIKGKGIANASSVEHYLSRVCYRRAAELFKNAAIASGLRSKIKS NFRLKELKNMKSGLPTTKSDNFPIPLVKQKGGQYTGFEISNHNSDFIIKIPFGRWQV KKEIDKYRPWEKFDFEQVQKSPKPISLLLSTQRRKRNKGWSKDEGTEAEIKKVMN GDYQTSYIEVKRGSKICEKSAWMLNLSIDVPKIDKGVDPSIIGGIAVGVRSPLVCAIN NAFSRYSISDNDLFHFNKKMFARRRILLKKNRHKRAGHGAKNKLKPITILTEKSERF RKKLIERWACEIADFFIKNKVGTVQMENLESMKRKEDSYFNIRLRGFWPYAEMQN KIEFKLKQYGIEIRKVAPNNTSKTCSKCGHLNNYFNFEYRKKNKFPHFKCEKCNFK ENAAYNAALNISNPKLKSTKERPAPKKKRKVGSATEFSRAMLPPNVIDTDFIDEEVL MSLVIEMGLDRIKELPELWLGQNEFDFMTDFVCKQQPSRVSCGGGGSPSGQISNQA LALAPSSAPVLAQTMVPSSAMVPLAQPPAPAPVLTPGPPQSLSAPVPKSTQAGEGTL SEALLHLQFDADEDLGALLGNSTDPGVFTDLASVDNSEFQQLLNQGVSMSHSTAE PMLMEYPEAITRLVTGSQRPPDPAPTPLGTSGLPNGLSGDEDFSSIADMDFSALLSQI SSSGQGGGGSGFSVDTSALLDLFSPSVTVPDMSLPDLDSSLASIQELLSPQEPPRPPE AENSSPDSGKQLVHYTAQPLFLLDPGSVDTGSNDLPVLFELGEGSYFSEGDGFAED PTISLLTGSEPPKAKDPTVS
**dCasMINI-CN: Cas12f (D326A/D510A/D143R/T147R/K330R/E528R), SV40 Nuclear Localization Sequence, CITED2-TAD, Glysine-Serine Linker Sequence, NCOA3-TAD, FOXO3-TAD, ZN473-KRAB**
MAKNTITKTLKLRIVRPYNSAEVEKIVADEKNNREKIALEKNKDKVKEACSKHLK VAAYCTTQVERNACLFCKARKLDDKFYQKLRGQFPDAVFWQEISEIFRQLQKQAA EIYNQSLIELYYEIFIKGKGIANASSVEHYLSRVCYRRAAELFKNAAIASGLRSKIKS NFRLKELKNMKSGLPTTKSDNFPIPLVKQKGGQYTGFEISNHNSDFIIKIPFGRWQV KKEIDKYRPWEKFDFEQVQKSPKPISLLLSTQRRKRNKGWSKDEGTEAEIKKVMN GDYQTSYIEVKRGSKICEKSAWMLNLSIDVPKIDKGVDPSIIGGIAVGVRSPLVCAIN NAFSRYSISDNDLFHFNKKMFARRRILLKKNRHKRAGHGAKNKLKPITILTEKSERF RKKLIERWACEIADFFIKNKVGTVQMENLESMKRKEDSYFNIRLRGFWPYAEMQN

KIEFKLKQYGIEIRKVAPNNTSKTCSKCGHLNNYFNFEYRKKNKFPHFKCEKCNFK<br>ENAAYNAALNISNPKLKSTKERPAPKKKRKVGSATEFSRAMLPPNVIDTDFIDEEVL<br>MSLVIEMGLDRIKELPELWLGQNEFDFMTDFVCKQQPSRVSCGGGGSEGQSDERA<br>LLDQLHTLLSNTDATGLEEIDRALGIPELVNQGQALEPKQGSGSGSHEKFPSDLDLD<br>MFNGSLECDMESIIRSELMDADGLDFNFDSGSGSGSFVTLKDVGMDFTLGDWEQL<br>GLEQGDTFWDTALDNCQDLFLL
**dCasMINI-CM: Cas12f (D326A/D510A/D143R/T147R/K330R/E528R), SV40 Nuclear Localization Sequence, CITED2-TAD, Glysine-Serine Linker Sequence, MRTF-A-TAD, STAT1-TAD, Neh4-Neh5-TAD**
MAKNTITKTLKLRIVRPYNSAEVEKIVADEKNNREKIALEKNKDKVKEACSKHLK<br>VAAYCTTQVERNACLFCKARKLDDKFYQKLRGQFPDAVFWQEISEIFRQLQKQAA<br>EIYNQSLIELYYEIFIKGKGIANASSVEHYLSRVCYRRAAELFKNAAIASGLRSKIKS<br>NFRLKELKNMKSGLPTTKSDNFPIPLVKQKGGQYTGFEISNHNSDFIIKIPFGRWQV<br>KKEIDKYRPWEKFDFEQVQKSPKPISLLLSTQRRKRNKGWSKDEGTEAEIKKVMN<br>GDYQTSYIEVKRGSKICEKSAWMLNLSIDVPKIDKGVDPSIIGGIAVGVRSPLVCAIN<br>NAFSRYSISDNDLFHFNKKMFARRRILLKKNRHKRAGHGAKNKLKPITILTEKSERF<br>RKKLIERWACEIADFFIKNKVGTVQMENLESMKRKEDSYFNIRLRGFWPYAEMQN<br>KIEFKLKQYGIEIRKVAPNNTSKTCSKCGHLNNYFNFEYRKKNKFPHFKCEKCNFK<br>ENAAYNAALNISNPKLKSTKERPAPKKKRKVGSATEFSRAMLPPNVIDTDFIDEEVL<br>MSLVIEMGLDRIKELPELWLGQNEFDFMTDFVCKQQPSRVSCGGGGSSSSQQMDD<br>LFDILIQSGEISADFKEPPSLPGKEKPSPKTVCGSPLAAQPSPSAELPQAAPPPPGSPS<br>LPGRLEDFLESSTGLPLLTSGHDGPEPLSLIDDLHSQMLSSTAILDHPPSPMDTSELH<br>FVPEPSSTMGLDLADGHLDSMDWLELSSGGPVLSLAPLSTTAPSLFSTDFLDGHDL<br>QLHWDSGSSEVHPSRLQTTDNLLPMSPEEFDEVSRIVGSVEFDSASSDALYFDDCM<br>QLLAQTFPFVDDNESGGGSGGSGSSQDIEQVWEELLSIPELQCLNIENDKLVE
**dCasMINI-MC: Cas12f (D326A/D510A/D143R/T147R/K330R/E528R), SV40 Nuclear Localization Sequence, MRTF-A-TAD, STAT1-TAD, Neh4-Neh5-TAD, Glysine-Serine Linker Sequence, CITED2-TAD**
MAKNTITKTLKLRIVRPYNSAEVEKIVADEKNNREKIALEKNKDKVKEACSKHLK<br>VAAYCTTQVERNACLFCKARKLDDKFYQKLRGQFPDAVFWQEISEIFRQLQKQAA<br>EIYNQSLIELYYEIFIKGKGIANASSVEHYLSRVCYRRAAELFKNAAIASGLRSKIKS<br>NFRLKELKNMKSGLPTTKSDNFPIPLVKQKGGQYTGFEISNHNSDFIIKIPFGRWQV<br>KKEIDKYRPWEKFDFEQVQKSPKPISLLLSTQRRKRNKGWSKDEGTEAEIKKVMN<br>GDYQTSYIEVKRGSKICEKSAWMLNLSIDVPKIDKGVDPSIIGGIAVGVRSPLVCAIN<br>NAFSRYSISDNDLFHFNKKMFARRRILLKKNRHKRAGHGAKNKLKPITILTEKSERF<br>RKKLIERWACEIADFFIKNKVGTVQMENLESMKRKEDSYFNIRLRGFWPYAEMQN<br>KIEFKLKQYGIEIRKVAPNNTSKTCSKCGHLNNYFNFEYRKKNKFPHFKCEKCNFK<br>ENAAYNAALNISNPKLKSTKERPAPKKKRKVGSATEFSRSSSQQMDDLFDILIQSGE<br>ISADFKEPPSLPGKEKPSPKTVCGSPLAAQPSPSAELPQAAPPPPGSPSLPGRLEDFLE<br>SSTGLPLLTSGHDGPEPLSLIDDLHSQMLSSTAILDHPPSPMDTSELHFVPEPSSTMG<br>LDLADGHLDSMDWLELSSGGPVLSLAPLSTTAPSLFSTDFLDGHDLQLHWDSGSSE<br>VHPSRLQTTDNLLPMSPEEFDEVSRIVGSVEFDSASSDALYFDDCMQLLAQTFPFV<br>DDNESGGGSGGSGSSQDIEQVWEELLSIPELQCLNIENDKLVEGGGGSAMLPPNVI<br>DTDFIDEEVLMSLVIEMGLDRIKELPELWLGQNEFDFMTDFVCKQQPSRVSC

**dCasMINI-VPR:** **Cas12f (D326A/D510A/D143R/T147R/K330R/E528R)**, **SV40 Nuclear Localization Sequence**, **VPR**
MAKNTITKTLKLRIVRPYNSAEVEKIVADEKNNREKIALEKNKDKVKEACSKHLK<br>VAAYCTTQVERNACLFCKARKLDDKFYQKLRGQFPDAVFWQEISEIFRQLQKQAA<br>EIYNQSLIELYYEIFIKGKGIANASSVEHYLSRVCYRRAAELFKNAAIASGLRSKIKS<br>NFRLKELKNMKSGLPTTKSDNFPIPLVKQKGGQYTGFEISNHNSDFIIKIPFGRWQV<br>KKEIDKYRPWEKFDFEQVQKSPKPISLLLSTQRRKRNKGWSKDEGTEAEIKKVMN<br>GDYQTSYIEVKRGSKICEKSAWMLNLSIDVPKIDKGVDPSIIGGIAVGVRSPLVCAIN<br>NAFSRYSISDNDLFHFNKKMFARRRILLKKNRHKRAGHGAKNKLKPITILTEKSERF<br>RKKLIERWACEIADFFIKNKVGTVQMENLESMKRKEDSYFNIRLRGFWPYAEMQN<br>KIEFKLKQYGIEIRKVAPNNTSKTCSKCGHLNNYFNFEYRKKNKFPHFKCEKCNFK<br>ENAAYNAALNISNPKLKSTKERPAPKKKRKVGSATEFEASGSGRADALDDFDLDM<br>LGSDALDDFDLDMLGSDALDDFDLDMLGSDALDDFDLDMLINSRSSGSPKKKRK<br>VGSQYLPDTDDRHRIEEKRKRTYETFKSIMKKSPFSGPTDPRPPPRRIAVPSRSSASV<br>PKPAPQPYPFTSSLSTINYDEFPTMVFPSGQISQASALAPAPPQVLPQAPAPAPAPAM<br>VSALAQAPAPVPVLAPGPPQAVAPPAPKPTQAGEGTLSEALLQLQFDDEDLGALLG<br>NSTDPAVFTDLASVDNSEFQQLLNQGIPVAPHTTEPMLMEYPEAITRLVTGAQRPPD<br>PAPAPLGAPGLPNGLLSGDEDFSSIADMDFSALLGSGSGSRDSREGMFLPKPEAGSA<br>ISDVFEGREVCQPKRIRPFHPPGSPWANRPLPASLAPTPTGPVHEPVGSLTPAPVPQP<br>LDPAPAVTPEASHLLEDPDEETSQAVKALREMADTVIPQKEEAAICGQMDLSHPPPR<br>GHLDELTTTLESMTEDLNLDSPLTPELNEILDTFLNDECLLHAMHISTGLSIFDTSLF

Table S4. Summary of predicted immunogenic peptides across TADs.

TADs	total # 9mer peptides	# 9mers score > 0	% 9mers score > 0
NFZ	134	59	44.03%
p65HSF1	305	104	34.10%
MSN	282	109	38.65%
CITED2	52	38	73.08%
NP fusion	452	169	37.39%
CP fusion	370	144	38.92%
CN fusion	199	101	50.75%
CM fusion	347	149	42.94%
VP64	42	21	50.00%
VPR	523	256	48.95%

Table S5. The correlation of RNAseq samples in this study. Numbers indicate Pearson's correlation coefficient.

	ctrl-1	ctrl-2	NP-1	NP-2	VPR-1	VPR_2
ctrl-1	1.00	1.00	0.97	0.99	0.99	0.99
ctrl-2	1.00	1.00	0.97	1.00	0.99	1.00
NP-1	0.97	0.97	1.00	0.98	1.00	0.98
NP-2	0.99	1.00	0.98	1.00	0.99	1.00
VPR-1	0.99	0.99	1.00	0.99	1.00	0.99
VPR_2	0.99	1.00	0.98	1.00	0.99	1.00

Table S6. The sgRNA guide sequences used in this study.

Gene	Sequences (5'-3')	Reference
EGFP	sg1: TCTCGATCTGTGCGCGACAT	This study
	sg2: CCTCGATCCTAGCGCGACAT	This study
	sg3: GTGCGCGACATCGGCTACGC	This study
	sg4: AGTGAGTTCTGATCGTGTCA	This study
	sg5: GGAGGAAGCAAGCGCGACAT	This study
	sg6: GTGGTATTACTGCGCGACAT	This study
	sg7: ACGCGCGACATCGGCTACGC	This study
	sg8: TGATCTACCTCGCGCGACAT	This study
TTN	sg1: CCTTGGTGAAGTCTCCTTTG	2
	sg2: ATGTTAAAATCCGAAAATGC	2
	sg3: GGGCACAGTCCTCAGGTTTG	2
	sg4: ATGAGCTCTCTTCAACGTTA	2
HBG	sg1: TGGTCAAGTTTGCCTTGTCA	3
	sg2: TATTTGCATTGAGATAGTGT	3
	sg3: GGAGAAGAAAACTAGCTAAA	3
	sg4: TCCCTGAACTTTTCAAAAAT	3
NEUROD1	sg1: AGGGGAGCGGTTGTCGGAGG	2
	sg2: ACCTGCCCATTTGTATGCCG	2
	sg3: AGGTCCGCGGAGTCTCTAAC	2
	sg4: TAGAGGGGCCGACGGAGATT	2
ASCL1	sg1: CGGGAGAAAGGAACGGGAGG	2
	sg2: AAGAACTTGAAGCAAAGCGC	2
	sg3: TCCAATTTCTAGGGTCACCG	2
	sg4: GTTGTGAGCCGTCCTGTAGG	2
IL1B	sg1: AATAAACTGAGATAATTCTC	3
	sg2: TCAACTGCACAACGATTGTC	3
	sg3: ACTTCTTTGTACTTAAGTTT	3

	sg4: CCCACACCCTCAATACAGAC	3
SOX2	sg1: GCCCCCTTTCATGCAAAACC	3
	sg2: GTGGCTGGCAGGCTGGCTCT	3
	sg3: AAACAGCACTAAGACTACGT	3
	sg4: GGGGTGGGGCAGGGCACAGT	3
RHOXF2	sg1: ACGCGTGCTCTCCCTCATC	2
	sg2: CGCGTGCTCTCCCTCATCC	2
	sg3: CTGTGGGTTGGGCCTGCTG	2
	sg4: GTGGGAGGGGGAGTAGGATG	2
NEUROG2	sg1: GGCGGTGGCGGGGGAGGAGG	2
	sg2: CAATGAAAAGAATAAGCCAG	2
	sg3: GGGAAAGGCGGTGAAGAAAG	2
	sg4: CGGAGCTGGCGAAGCCGCAG	2
HBG	dCasMINI sg1: CATTGAGATAGTGTGGGGAAGGG	4
ASCL1	dCasMINI sg1: CAAGGAGCGGGAGAAAGGAACGG	4
CYC1p-mCherry	ACTTTAGTGCTGACACATAC	7

Table S7. The qRT-PCR primers used in this study.

Gene	Primers (5’-3’)	Reference
HBG	F: GCTGAGTGAACTGCACTGTGA<br>R: GAATTCTTTGCCGAAATGGA	4
TTN	F: TGTTGCCACTGGTGCTAAAG<br>R: ACAGCAGTCTTCTCCGCTTC	5
NEUROD1	F: GGATGACGATCAAAAGCCCAA<br>R: GCGTCTTAGAATAGCAAGGCA	5
ASCL1	F: GGGCTCTTACGACCCGCTCA<br>R: AGGTTGTGCGATCACCCTGCTT	4
SOX2	F: ACAGCAAATGACAGCTGCAAA<br>R: TCGGCATCGCGGTTTTT	3
IL1B	F: ATGATGGCTTATTACAGTGGCAA<br>R: GTCGGAGATTCGTAGCTGGA	3
RHOXF2	F: TTTTCCAACGCGAGCAGTTC<br>R: GGCAGCATGTTTCTTGCCAT	6
NEUROG2	F: TGGGTCTGGTACACGATTGC<br>R: GGGTCTCGATCTTGGTGAGC	2
GAPDH	F: CAATGACCCCTTCATTGACC<br>R: TTGATTTTGGAGGGATCTCG	4

References

1. Calis, J. J. A.; Maybeno, M; Greenbaum, J. A.; et al Properties of MHC Class I Presented Peptides That Enhance Immunogenicity. *PLoS Comput Biol* **2013** *9* (10), e1003266.
2. Chavez, A.; Scheiman, J.; Vora, S.; Pruitt, B. W.; Tuttle, M.; P R Iyer, E.; Lin, S.; Kiani, S.; Guzman, C. D.; Wiegand, D. J.; Ter-Ovanesyan, D.; Braff, J. L.; Davidsohn, N.; Housden, B. E.; Perrimon, N.; Weiss, R.; Aach, J.; Collins, J. J.; Church, G. M. Highly Efficient Cas9-Mediated Transcriptional Programming. *Nat. Methods* **2015**, *12* (4), 326–328.
3. Liu, J.; Chen, Y.; Nong, B.; Luo, X.; Cui, K.; Li, Z.; Zhang, P.; Tan, W.; Yang, Y.; Ma, W.; Liang, P.; Songyang, Z. CRISPR-Assisted Transcription Activation by Phase-Separation Proteins. *Protein Cell* **2023**, *3* (1), 29–35.
4. Xu, X.; Chemparathy, A.; Zeng, L.; Kempton, H. R.; Shang, S.; Nakamura, M.; Qi, L. S. Engineered Miniature CRISPR-Cas System for Mammalian Genome Regulation and Editing. *Mol. Cell* **2021**, *81* (20), 4333–4345.
5. Chavez, A.; Tuttle, M.; Pruitt, B. W.; Ewen-Campen, B.; Chari, R.; Ter-Ovanesyan, D.; Haque, S. J.; Cecchi, R. J.; Kowal, E. J. K.; Buchthal, J.; Housden, B. E.; Perrimon, N.; Collins, J. J.; Church, G. Comparison of Cas9 Activators in Multiple Species. *Nat. Methods* **2016**, *13* (7), 563–567.
6. Perez-Pinera, P. et al. RNA-guided Gene Activation by CRISPR-Cas9–based Transcription Factors. *Nat. Methods* **2013**, *10* (10), 973–976.
7. Lian, J.; HamediRad, M.; Hu, S.; Zhao, H. Combinatorial Metabolic Engineering Using An Orthogonal Tri-functional CRISPR System. *Nat. Commun.* **2017**, *8*, 1688.
